# FtsEX-independent control of RipA-mediated cell separation in *Corynebacteriales*

**DOI:** 10.1073/pnas.2214599119

**Published:** 2022-12-05

**Authors:** Quentin Gaday, Daniela Megrian, Giacomo Carloni, Mariano Martinez, Bohdana Sokolova, Mathilde Ben Assaya, Pierre Legrand, Sebastien Brûlé, Ahmed Haouz, Anne Marie Wehenkel, Pedro M. Alzari

**Affiliations:** ^a^Unité de Microbiologie Structurale, Institut Pasteur, CNRS, Université Paris Cité, Paris 75015, France; ^b^Synchrotron SOLEIL, 91192 Gif-sur-Yvette, France; ^c^Plate-forme de biophysique moléculaire, Centre de Ressources et de Recherches Technologiques, Institut Pasteur, CNRS, F-75015 Paris, France; ^d^Plate-forme de cristallographie, Centre de Ressources et de Recherches Technologiques, Institut Pasteur, CNRS, F-75015, Paris, France

**Keywords:** bacterial cell division, peptidoglycan, Corynebacterium glutamicum, structural biology

## Abstract

The bacterial cell wall is a multi-layered mesh, whose major component is peptidoglycan (PG), a sugar polymer cross-linked by short peptide stems. During cell division, a careful balance of PG synthesis and degradation, precisely coordinated both in time and space, is necessary to prevent uncontrolled destruction of the cell wall. In *Corynebacteriales,* the D,L endopeptidase RipA has emerged as a major PG hydrolase for cell separation, and RipA defaults have major implications for virulence of the human pathogens *Mycobacterium tuberculosis* and *Corynebacterium diphtheriae*. However, the precise mechanisms by which RipA mediates cell separation remain elusive. Here we report phylogenetic, biochemical, and structural analysis of the *Corynebacterium glutamicum* homologue of RipA, Cg1735. The crystal structures of full-length Cg1735 in two different crystal forms revealed the C-terminal NlpC/P60 catalytic domain obtruded by its N-terminal conserved coiled-coil domain, which locks the enzyme in an autoinhibited state. We show that this autoinhibition is relieved by the extracellular core domain of the transmembrane septal protein Cg1604. The crystal structure of Cg1604 revealed a (β/α) protein with an overall topology similar to that of receiver domains from response regulator proteins. The atomic model of the Cg1735–Cg1604 complex, based on bioinformatical and mutational analysis, indicates that a conserved, distal-membrane helical insertion in Cg1604 is responsible for Cg1735 activation. The reported data provide important insights into how intracellular cell division signal(s), yet to be identified, control PG hydrolysis during RipA-mediated cell separation in *Corynebacteriales*.

## Significance

Cell wall synthesis, maintenance, and degradation are essential processes for bacteria and important targets for drug development. The enzymes responsible for peptidoglycan hydrolysis (termed *murein hydrolases*) must be tightly regulated to avoid autolysis. In the order *Corynebacteriales*, which includes the important pathogens *Mycobacterium tuberculosis* and *Corynebacterium diphtheriae, *the most important murein hydrolase for cell separation is the endopeptidase RipA. We present here the crystal structure of a full-length RipA homologue from *Corynebacterium glutamicum, *Cg1735*, *which reveals an unusual mode of autoinhibition. We describe how this autoinhibition is relieved by the conserved transmembrane protein Cg1604 and propose an FtsEX-independent model for septal control of PG hydrolysis *via* the Cg1604–Cg1735 complex.

The *Corynebacteriales* order of bacteria includes many significant human pathogens, such as *Mycobacterium tuberculosis* (*Mtb*) and *Corynebacterium diphtheriae*. These organisms, often referred to as mycolates, display an atypical cell envelope architecture. Their peptidoglycan (PG) cell wall matrix is uniquely decorated with arabinogalactan polymers that covalently anchor an outer surface layer—the mycomembrane—primarily made up of mycolic acids (1). Long viewed as a relatively inert barrier to osmotic lysis, the PG sacculus is now known to form a dynamic structure that undergoes constant growth, remodeling, and partitioning during the cell cycle (2). Despite extensive biochemical and genetic characterization of the enzymes responsible for the synthesis and degradation of PG (3–5), the mechanisms by which these enzymes coordinate their activities and synchronize them with the cell cycle status remain poorly defined. During cytokinesis, the PG layer is synthesized continuously at the septal junction (6) and needs to be hydrolyzed at precisely the right time and place to allow daughter cell separation (V-snapping). The PG hydrolases essential for this process must be tightly regulated to avoid toxicity (7), since lack of activation leads to multi-septate elongated cells (8) and conversely their dysregulation can be lethal to bacteria (9).

PG hydrolase genes usually show high redundancy, and many of these enzymes form multi-protein complexes in which divisome proteins control the hydrolytic activity through proteolysis, protein–protein interactions, or allosteric regulation (10–12). Despite their importance, limited information is available on the PG hydrolases essential for cell division in *Corynebacteriales*. In *Mtb*, possibly one of the best studied organisms in this respect, the major PG hydrolase involved in cell separation is Rv1477, a member of the NlpC/P60 superfamily that is also called RipA (for Rpf-interacting protein A) (13, 14). RipA depletion results in the formation of elongated multi-septated cells in vitro, both in *Mtb* and *Mycobacterium smegmatis* (14, 15), and leads to reduced *Mtb* replication in macrophages and to clearance of *Mtb* in infected mice (15). In *Corynebacterium glutamicum (Cglu)*, another NlpC/P60 superfamily member, Cg1735, was found to fulfill a similar role. Disruption of cg1735 resulted in elongated cells with multiple septa (16, 17), a morphological phenotype that was not observed upon disruption of any of the three other genes (cg0784, cg2401, or cg2402) coding for NlpC/P60 proteins in the genome (16). A similar defect in cell separation involving the formation of chains of bacteria was observed in *C. diphtheriae* upon disruption of the Cg1735 homolog DIP1281 (18) and in *M. marinum* upon disruption of IipA (19). Here we show that all these NlpC/P60 hydrolases essential for cell separation are indeed part of a single family in *Corynebacteriales* containing *Mtb* RipA. We describe the full-length crystal structure of one of them, *Cglu* Cg1735, which reveals a striking arrangement of the N-terminal coiled-coil domain tightly associated to the C-terminal NlpC/P60 catalytic domain (CD). We demonstrate that this autoinhibition is released by the membrane-bound periplasmic protein Cg1604 and map the site of interaction. Together with phylogenetic analysis, biochemical, and biophysical characterization, this structural study provides new important insights into the mode of action of the RipA family of endopeptidases on cell separation in *Corynebacteriales*.

## Results

### Cg1735 Belongs to the RipA Family of PG Hydrolases.

**Fig. 1.**
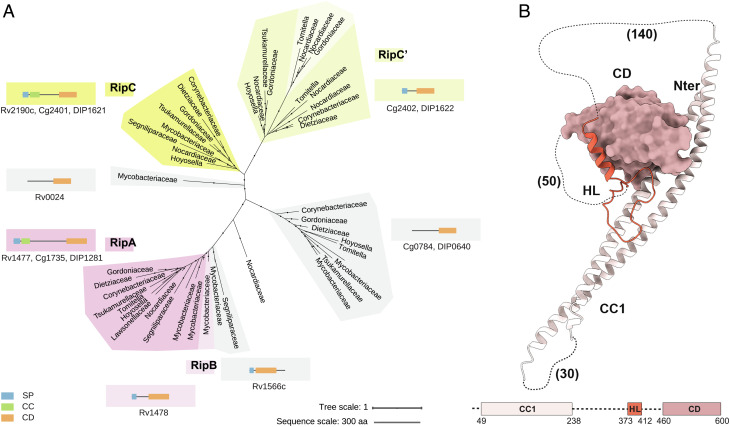
Phylogenetic distribution and overall structure of the RipA homolog Cg1735. (*A*) Phylogeny of the NlpC/P60 superfamily in *Corynebacteriales*. Clades RipA (dark pink), RipB (light pink), and RipC (dark yellow) were labeled according to *Mtb* nomenclature. Clade RipC’ (light yellow) corresponds to a duplication absent in *Mtb*. Other duplications are indicated in gray. A schematic representation of the domain organization of each NlpC/P60 protein family is indicated for each clade (SP: signal peptide, CC: coiled-coil, and CD: catalytic domain). Locus tags of the corresponding proteins in *Mtb* (Rv), *Cglu* (Cg), and *C. diphtheriae* (DIP) are indicated for each clade*.* Monophyletic families were collapsed into a single branch for clarity (for the full version, see *SI Appendix,* Fig. S1). Dots indicate UFB  ≥ 0.85. (*B*) Overall structure of full-length Cg1735 in the orthorhombic crystal form. The structural domains of the protein (CC1, HL, and CD) are shown in different shades and missing connections are indicated (length in amino acids in parenthesis). The domains are represented below in linear form (dotted lines represent disordered regions of the sequence).

Members of the NlpC/P60 superfamily in *Corynebacteriales* are highly redundant. *Corynebacteriaceae* genomes usually contain four genes, while most *Mycobacteriaceae* contain five, and *Nocardiaceae* genomes may contain more than eight genes (*SI Appendix,* Table S1). A phylogenetic study of the NlpC/P60 superfamily in the *Corynebacteriales* order (Fig. 1*A* and *SI Appendix,* Fig. S1) revealed that *Cglu* cg1735 belongs to a major clade that includes homologous enzymes also known to be crucial for cell separation in other bacteria, such as *Mtb* Rv1477 (RipA) and *C. diphtheriae* DIP1281. Most proteins in this group, which we will here refer to as the RipA family, are found in members from all *Corynebacteriales*. Within the *Mycobacteriaceae* family (but not in other *Corynebacteriales*), these genes have undergone a duplication event leading to a pair of syntenic genes, as seen for instance in *Mtb* with Rv1477 (RipA) and Rv1478 (RipB). Multiple sequence alignment of the RipA family (*SI Appendix*, Fig. S2*A*) reveals two highly conserved regions, an N-terminal coiled-coil domain and a C-terminal NlpC/P60 CD, which are connected to each other by a highly variable linker both in sequence and length (from ~230 residues in most *Mycobacteriaceae* to ~350 residues in most *Corynebacteriaceae*).

A second, phylogenetically distant, clade in the tree also includes similar two-domain enzymes (Fig. 1*A*). This group includes NlpC/P60 enzymes such as *Mtb* Rv2190c (RipC) and *Cglu* Cg2401, both of which were found to play no major role in cell separation (16, 20). The domain organization of most enzymes from this group also consists of a highly conserved N-terminal coiled-coil linked to the CDs (*SI Appendix*, Fig. S2*B*), but the interdomain region is shorter (~150 residues) and displays a lower variability in length. Interestingly, an independent gene duplication event (RipC’) is also observed in this group for all *Corynebacteriales* except *Mycobacteriaceae* and *Segniliparaceae*, and in some species the duplicated genes are syntenic (as in *Cglu* cg2401 and cg2402, and *C. diphtheriae* DIP1621 and DIP1622).

### Overall Structure of Cg1735.

The full periplasmic region of Cg1735 (residues 20 to 600) was crystallized in two different crystal forms with a high (80 to 85%) solvent content. The trigonal form was solved by single-wavelength anomalous diffraction (SAD) techniques at 3.5 Å resolution and the orthorhombic form by molecular replacement at 4.5 Å resolution (see *Materials and Methods* and *SI Appendix,* Table S2). Both structures reveal a tight complex (Fig. 1*B*) between the C-terminal NlpC/P60 CD (residues 460 to 600) and an anti-parallel two-helical coiled-coil domain (CC1, residues 46 to 238) corresponding to the highly conserved N-terminal region in the RipA family (*SI Appendix,* Fig. S2*A*). In addition, a small helix-loop (HL) domain (residues 373 to 412) folds into an α-helix packed against the CD followed by a loop that embraces the coiled-coil helices (Fig. 1*B*). The CC1-HL-CD complexes are very similar in the two crystal forms (*SI Appendix,* Fig. S3), with an rmsd of 0.9 Å for 278 equivalent Cα positions. Although the HL domain is not part of the NlpC/P60 domain, its tight association suggests that it forms integral part of the catalytic core. Interestingly, a helix associated to the CD was also found at an equivalent position in the crystal structures of *Mtb* RipA and RipB (10, 21) (*SI Appendix,* Fig. S4), but not in other NlpC/P60 structures. However, in the two *Mtb* structures, the helix is preceded by an extended region that binds to the active site in the crystal, whereas in Cg1735 it is the N-terminal coiled-coil that occupies and blocks the catalytic cleft (see below).

In the orthorhombic crystal form, the long interconnecting linker (residues 239 to 372) is present but disordered in the crystal. Instead, crystal contacts in the trigonal form promote a disorder-to-order transition in this region that extends the short HL helix into a full anti-parallel two-helical coiled-coil domain, CC2 (residues 243 to 386) (*SI Appendix,* Fig. S5*A*). The presence of this new structural element precludes complex formation between the CD and the CC1 domain to occur within the same monomer. Instead, the CD–CC1 complex now forms between two different monomers in the asymmetric unit (*SI Appendix,* Fig. S5*B*). Analytical ultracentrifugation experiments at different protein concentrations showed that full-length RipA behaves as a monomer in solution, with no trace of dimer formation even at 5 mg/mL, the highest tested concentration (*SI Appendix,* Fig. S5*C*), demonstrating that dimerization is due to crystallization-induced domain-swapping effects. The CC2 domain is deeply engaged in—and stabilized by—crystal packing contacts in the trigonal space group (*SI Appendix,* Fig. S6). Poorly conserved in RipA homologs (*SI Appendix,* Fig. S2*A*), the CC2 sequence region is rich in poly-alanine and poly-glutamine segments (63% of the 144 residues are Ala or Gln), which share a common propensity to form α-helical coiled-coil structures (22, 23). It therefore appears that protein–protein interactions can induce an extensive conformational transition from random coil to helical structure that, depending on the molecular context, may lead to the formation of a second coiled-coil domain (as seen in the trigonal crystal structure), or to a single α-helical expansion of the conserved CC1 domain (*SI Appendix,* Fig. S7). It cannot be excluded that one or more of these coiled-coils structures induced by protein–protein interactions could play some functional role in a cellular context.

### Cg1735 Reveals an Autoinhibited Conformation in the Crystals.

**Fig. 2.**
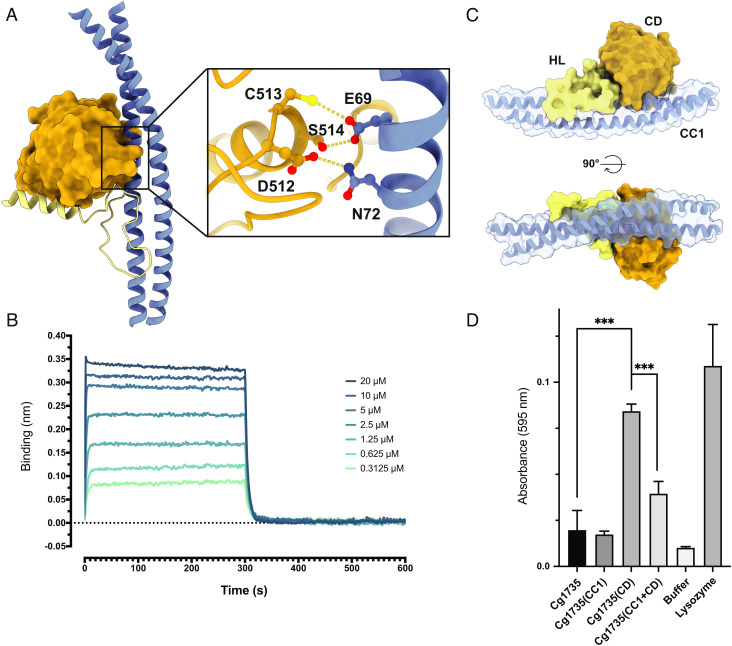
The crystal structure reveals an autoinhibited conformation of Cg1735. (*A*) The CD (orange surface) and the associated HL domain (light yellow cartoon) form an intramolecular complex with the CC1 domain (blue cartoon) that blocks the catalytic cleft and inactivates Cg1735. The inset shows the hydrogen bonding interactions of the catalytic cysteine (C513) and the two adjacent residues with two strictly conserved residues, Glu69 and Asn72, from the coiled-coil domain. (*B*) Sensorgrams of Cg1735_CD_ binding to immobilized Cg1735_CC1_ by biolayer interferometry. A series of measurements using a range of concentrations for Cg1735_CD_ was performed to derive equilibrium dissociation constant (*K*_d_) value (*SI Appendix,* Fig. S8). (*C*) The HL domain (light yellow) further stabilizes the autoinhibited conformation by interacting extensively with the CD (orange) and the CC1 (blue) domains. (*D*) Endopeptidase activity tests of the CD alone (Cg1735_CD_) on RBB-PG in the presence or absence of the CC1 domain (Cg1735_CC1_). The activities of full-length Cg1735 and lysozyme were measured as controls. Asterisks represent the statistical significance (*P*- values) between the difference of the means using unpaired *t *tests with Prism software (*P < 0.05; **P < 0.01; ***P < 0.001).

The structure of the NlpC/P60 CD is very similar to those of the *Mtb* homologues RipA and RipB (10, 21). The overall rmsd is 1.02/0.99 Å for 134/130 aligned residues, respectively, and most structural differences are clustered in two external loops of the protein. The high conservation of the active site cleft suggests that Cg1735 shares the substrate specificity of the mycobacterial homologs, which cleave PG fragments between the D-glutamate and the meso-diaminopimelate stem residues (21). In the full-length RipA structures, however, access to the active site is blocked by its interaction with the coiled-coil domain CC1 (Fig. 2*A*). The catalytic cysteine, Cys513, and the two adjacent residues, Asp512 and Ser514, form hydrogen bonding interactions with two amino acid residues from the coiled-coil helix, Glu69 and Asn72, both of which are conserved in the whole RipA family. Using separate constructs for the CC1 domain (Cg1735_CC1_, residues 20 to 238) and the CD (Cg1735_CD_, residues 460 to 600), we measured an apparent dissociation constant *Kd* of 1.2 ± 0.2 μM by bio-layer interferometry (BLI) (Fig. 2*B* and *SI Appendix,* Fig. S8), indicating that intramolecular complex formation is a genuine feature of the protein in solution and not a consequence of crystallization. The binding strength is even higher in the context of the full-length protein, because the Cg1735_CC1_–Cg1735_CD_ interaction is further stabilized by the HL domain that binds on top of the other two domains, burying a total molecular surface of ~3,150 Å^2^ (Fig. 2*C*). These interactions block substrate access to the active site, locking the CD in an inactive conformation. This autoinhibition mechanism was further confirmed by activity assays of Cg1735_CD_ on purified PG. We found that, even in the absence of the HL domain, addition of Cg1735_CC1_ to the reaction mixture significantly decreased the constitutive activity of Cg1735_CD_ alone (Fig. 2*D*). In the same experiment, full-length Cg1735 was inactive, strongly suggesting that the autoinhibited state is prevalent in solution.

### The Septal Transmembrane (TM) Protein Cg1604 Activates Cg1735.

**Fig. 3.**
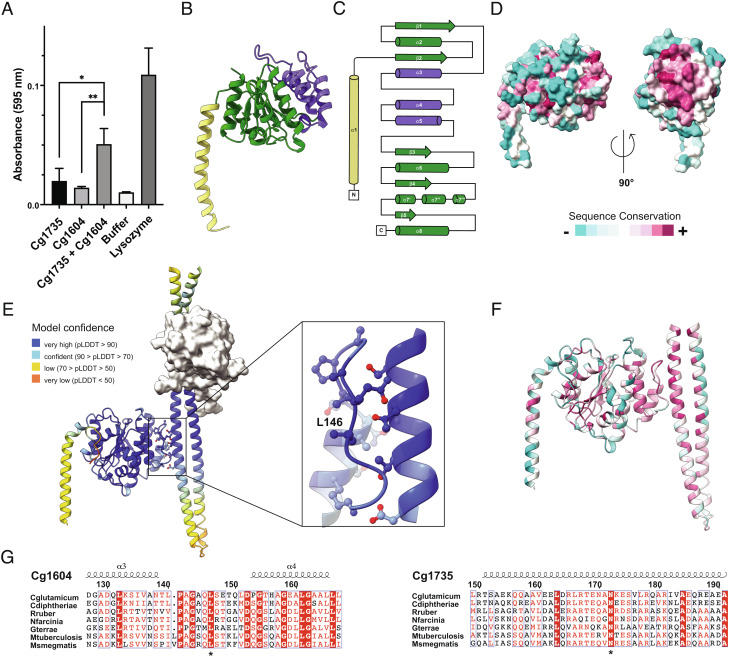
The septal TM protein Cg1604 activates Cg1735. (*A*) Activity assays of Cg1735 in the presence or absence of Cg1604. The activity of lysozyme was measured as control. *P*-values were calculated as in Fig. 2*D*. (*B*) Crystal structure of the globular domain of Cg1604. (*C*) Secondary structure topology. (*D*) Mapping of conserved residues on the molecular surface of Cg1604. (*E*) AlphaFold-predicted complex between Cg1604 and Cg1735 (*SI Appendix,* Fig. S9). The *Inset* shows a zoom of the conserved interaction interface from the two proteins, with Leu146 at the center of this hydrophobic region. (*F*) Same as (*E*) but color-coded according to sequence conservation in Cg1604 and Cg1735 homologues in *Corynebacteriales*. (*G*) Multiple alignment of the interacting regions in both proteins for selected RipA homologs. The two highly conserved residues discussed in the text, Cg1604 Leu146 and Cg1735 Asn172, are indicated with an asterisk below the sequences.

The autoinhibited conformation of Cg1735 and the high dissociation constant of the intramolecular association pointed to the existence of specific regulatory protein(s) that could activate the hydrolase by promoting complex dissociation through protein–protein interactions. In principle, a possible candidate for such a role could be the FtsEX complex, which is known to use a similar mechanism to control cell wall hydrolysis in other species (24, 25). However, in *Mtb* FtsX was found to regulate RipC, but not the Cg1735 homologue RipA (20). Other potential candidates were Cg1603 and Cg1604, two TM proteins identified recently by analyzing *Cglu* transposon mutants sensitive to ethambutol (17). These proteins were found to form a complex at the division site and proposed to be part of the cell separation pathway in *Cglu* (17). We thus produced the soluble periplasmic domain of Cg1604 (Cg1603 has no extra-cytoplasmic globular domain) and assessed its effect on a PG activity assay of full-length Cg1735. As shown in Fig. 3*A*, a clear activation effect is observed upon addition of the Cg1604 periplasmic domain to the reaction mixture.

To elucidate the activation mechanism, we solved the crystal structure of the periplasmic domain of Cg1604 (residues 36 to 295) by SAD techniques at 2 Å resolution (see *Materials and Methods* and *SI Appendix,* Table S2). The structure revealed an extended N-terminal α-helix that connects the N-terminal TM region of the protein (absent in the construct) to a globular domain consisting of a parallel five-stranded β-sheet surrounded by α-helices on both sides (Fig. 3*B*). This domain displays the (β/α)_5_ topology characteristic of receiver domains from bacterial response regulators, except for the insertion of a helical subdomain between β-strands 2 and 3 (Fig. 3*C*). The globular core of Cg1604 has no obvious groove or pocket that might suggest a putative functional site. However, a patch of surface residues within the subdomain insertion (in the membrane distal face of the protein) is highly conserved in Cg1604 homologs (Fig. 3*D*), suggesting that this region might represent a functionally conserved binding site. Indeed, calculations with AlphaFold (26) on the two full-length proteins, Cg1735 and Cg1604, predicted with a relatively high confidence that the Cg1604 subdomain interacts with the CC1 domain of RipA (Fig. 3*E* and *SI Appendix,* Fig. S9), to form a complex in which the protein–protein interface is highly conserved in both proteins (Figs. 3 *F* and *G*). Intermolecular contacts involve helix α3 and its adjacent loop from Cg1604 and the second helix from the CC1 domain of Cg1735. The complex is mainly stabilized through hydrophobic interactions, with Cg1604 Leu146 occupying the center of the interface (Fig. 3 *E*, *Inset*), and several hydrogen bonding interactions between Cg1735 Asn172 and the main chain of Cg1604 (Figs. 3 *F* and *G*). Interestingly the Cg1735 coiled-coil helix in contact with Cg1604 (CC1 α2) is complementary to that blocking the catalytic cleft (CC1 α1).

**Fig. 4.**
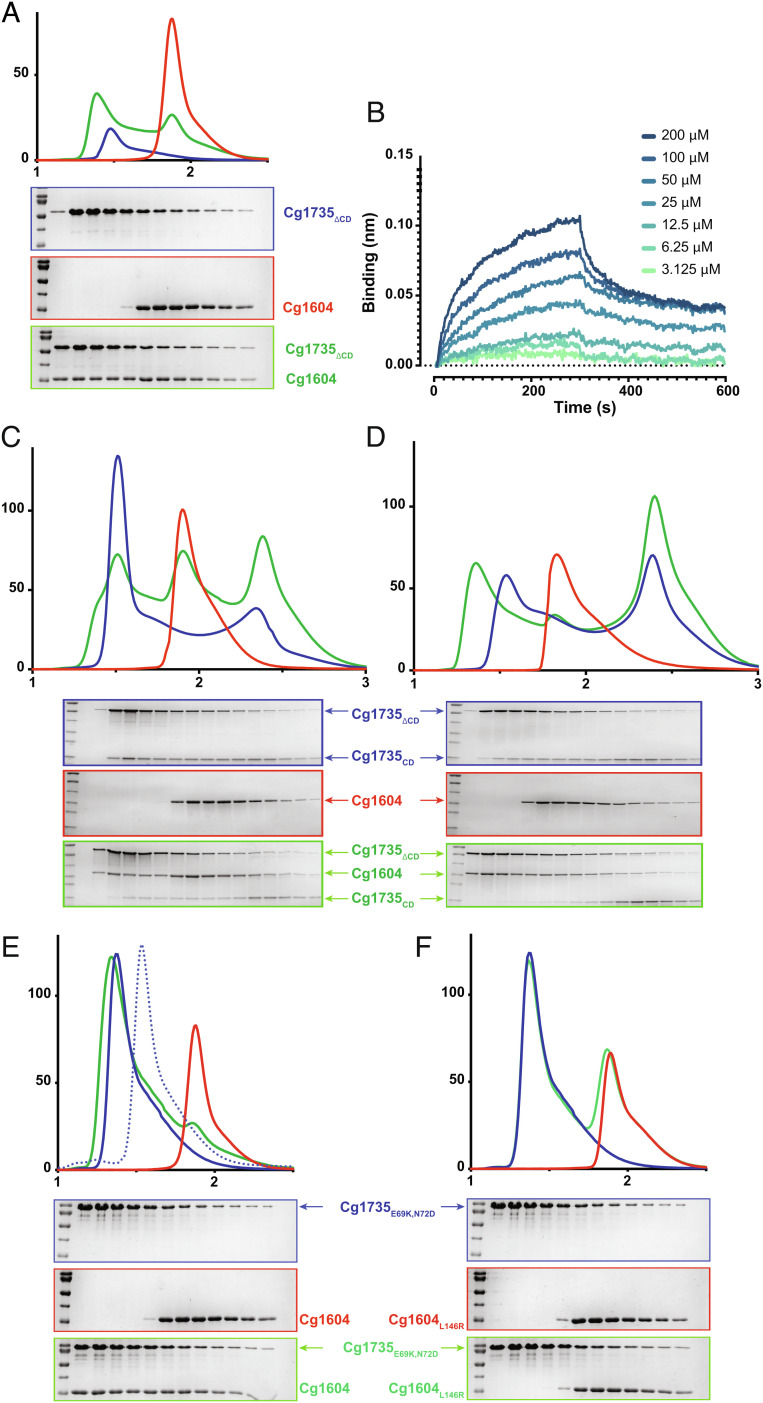
Cg1604–Cg1735 binding studies. All SEC experiments were run on a Superdex 200 Increase 5/150 column and SDS-PAGE gels were run for the same equivalent fractions of the chromatogram for each run. The units are the same for the x-axes (elution volume, mL) and y-axes (absorbance, mili-absorbance unit [mAU]) in all SEC experiments. Blue curves correspond to Cg1735 constructs, red curves correspond to Cg1604 constructs, and green curves correspond to the analyzed mixtures as described in each SDS-PAGE panel. (*A*) Cg1735_ΔCD_ and Cg1604 interact as shown by the leftmost shift of the green curve and the SDS-PAGE profile. (*B*) Sensorgrams of Cg1604 binding to immobilized Cg1735_ΔCD_ by biolayer interferometry. A series of measurements using a range of concentrations for Cg1604 was carried out to derive equilibrium dissociation constant (*K*_d_) value (*SI Appendix,* Fig. S10*A*). (*C*) Co-incubation at pH 7.5 of Cg1735_ΔCD_, Cg1735_CD_, and Cg1604 leads to partial interaction between Cg1735_ΔCD_ and Cg1604. (*D*) Co-incubation at pH 5 of Cg1735_ΔCD_, Cg1735_CD_, and Cg1604 leads to full interaction between Cg1735_ΔCD_ and Cg1604 and full dissociation between Cg1735_ΔCD_ and Cg1735_CD_. (*E*) Cg1604, which was unable to bind wild-type Cg1735, does bind the Cg1735_E69K, N72D_ double mutant. The elution profile of wild-type Cg1735 (dotted line) is also shown for comparison purposes. (*F*) The point mutant Cg1604_L146R_ does not interact with Cg1735_E69K, N72D_. The elution profiles and SDS-PAGE of Cg1604 alone in (*A*) and (*E*), and those for Cg1735_E69K, N72D_ alone in (*E*) and (*F*), correspond to the same experiment in each case.

In apparent contradiction with the above model, however, we failed to detect binding of Cg1604 to full-length Cg1735 using size exclusion chromatography (SEC). Given the strong intramolecular association of the CD with the coiled-coil domain, we reasoned that the latter could be locked in a conformation that was unable to bind Cg1604, at least under the conditions used in our experiments at neutral pH. In agreement with this hypothesis, Cg1604 was able to bind a Cg1735 construct lacking the CD (Cg1735_ΔCD_, residues 20 to 386) under the same experimental conditions (Fig. 4*A*), with an apparent *Kd* of 56 ± 10 μM as determined by BLI (Fig. 4*B* and *SI Appendix,* Fig. S10). The addition of Cg1604 partially dissociated the Cg1735_ΔCD_–Cg1735_CD_ complex, leading to an equilibrium between two distinct binary complexes in which Cg1735_ΔCD_ interacts with either the CD or Cg1604 (Fig. 4*C*). Moreover, when the same experiments were performed at pH 5, the equilibrium was clearly shifted toward formation of the Cg1735_ΔCD_–Cg1604 complex (Fig. 4*D*). From these experiments, we can propose a plausible activation mechanism, according to which Cg1604 binding shifts the equilibrium toward the active form of Cg1735 by promoting dissociation (or precluding formation) of the intramolecular CC1–CD complex, thus releasing the catalytic cleft for hydrolysis.

To further validate the protein–protein interaction model, we introduced two amino acid substitutions in Cg1735 (Glu69-Lys and Asn72-Asp) aimed at disrupting the intramolecular association of the CC1 domain with the CD (Fig. 2 *A*, *Inset*), to produce the constitutively open protein Cg1735_E69K, N72D_, and the point mutation Leu146-Arg in the Cg1604 interface (Cg1604_L146R_) aimed at disrupting the formation of the Cg1735–Cg1604 activation complex (Fig. 3 *E*, *Inset*). The far-UV circular dichroism (CD) spectra of these mutant proteins were very similar to those of the respective wild-type proteins (*SI Appendix,* Fig. S11), indicating that no significant changes in secondary structure resulted from the amino acid substitutions. As predicted by our model, Cg1735_E69K, N72D_ has a SEC profile consistent with a more elongated protein conformation than wild-type Cg1735 (Fig. 4*E*), because the mutated coiled-coil was unable to bind the active site cleft. Conversely to wild-type Cg1735, Cg1735_E69K, N72D_ interacted strongly with its activator Cg1604 under the same conditions (Fig. 4*E*). This interaction was completely abolished by the amino acid substitution Leu146-Arg on Cg1604 (Fig. 4*F*), further confirming that formation of the Cg1604–Cg1735 complex is mediated by the conserved molecular patch of residues in Cg1604 (Fig. 3*D*).

### Control of PG Hydrolysis during Cell Separation in Corynebacteriales.

**Fig. 5.**
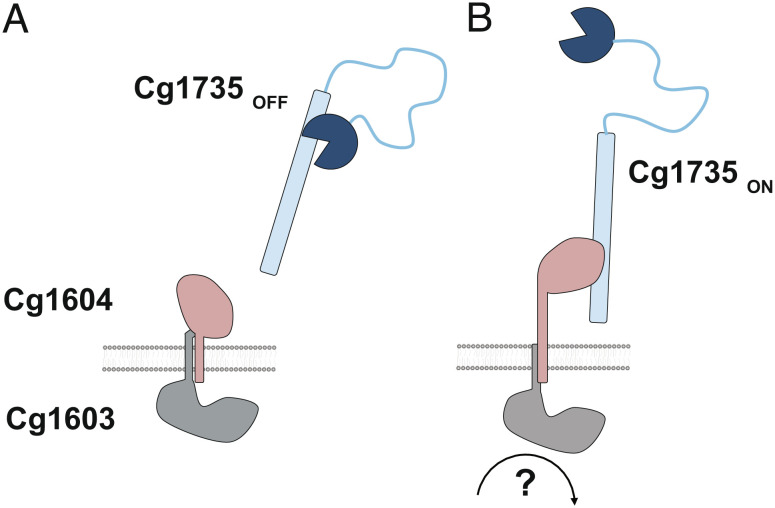
Model for Cg1604-mediated regulation of PG hydrolysis during cell separation in *Corynebacteriales*. (*A*) In the absence of a signal (yet to be identified) from the divisome, the septal Cg1603/Cg1604 complex restrains the Cg1604 extracellular domain, precluding its association with autoinhibited Cg1735. (*B*) Upon signal sensing, a conformational change of the Cg1603/Cg1604 complex releases the Cg1604 extracellular domain for interaction with Cg1735, unlocking in turn the CD for PG hydrolysis.

The above structural and biochemical studies support a working model for Cg1604-mediated activation of Cg1735, the major PG hydrolase responsible for cell separation in *Cglu* (Fig. 5). In this model, two septal TM proteins, Cg1603, with a cytoplasmic N-terminal globular core, and Cg1604, with a C-terminal globular core exposed to the cell envelope, form a signaling complex that couples intracellular cell division events with PG hydrolysis during the last stages of cell separation. Previous work showed that all three proteins localize to the septum, mutants defective for any of them showed a delayed V-snapping and led to the same ethambutol-sensitive phenotype, and the two TM proteins, Cg1603 and Cg1604, interact with each other (possibly through their TM segments), as shown using the POLAR two-hybrid approach (17). The experimental structure of Cg1603 is not available, but the AlphaFold model of its globular core predicts two distinct structural domains connected by a long α-helix. The protein is annotated as a putative thiamine pyrophosphokinase based on partial sequence homology, but the structural model argues against this hypothesis because the putative substrate binding sites on Cg1603 are either missing (thiamine) or very different (adenosine triphosphate [ATP]).

The activity and binding assays described above point to the interaction between Cg1604 and the CC1 domain of Cg1735 as a primary determinant of enzyme activation. However, the CC1 domain also binds the CD with an even higher affinity (*SI Appendix,* Fig. S10), and we were unable to observe Cg1604–Cg1735 complex formation in SEC experiments, suggesting that the ternary complex of RipA with the whole Cg1603–Cg1604 TM complex would likely be required in a physiological context for releasing the CD and triggering full activation. The actual nature of the triggering signal remains unknown. The partial structural similarity of Cg1603 to TPPKs leaves open the possibility that a putative ATPase activity of Cg1603 could serve as a driving force. Alternatively, since both Cg1603 and Cg1604 have a septal localization, a possible link to the divisome might be mediated by their respective TM domains and/or the globular cytoplasmic domain of Cg1603 via protein–protein interactions. Consistent with this hypothesis, the Cg1603 homolog in *M. smegmatis* (MSMEG_3748) and *Mtb* (Rv1698) were recently found in immunoprecipitations targeting the divisomal proteins FtsQ and FtsB, respectively (27, 28). Importantly, the wide phylogenetic distribution of Cg1603, Cg1604, and Cg1735 (17) and the sequence conservation of the intermolecular binding sites in both Cg1604 and Cg1735 homologs (Fig. 3*G*) strongly support the hypothesis of a conserved ancestral mechanism of PG hydrolysis regulation within the RipA family (Fig. 1*A*) in *Corynebacteriales*.

## Discussion

The high redundancy of PG hydrolases and the fact that single-enzyme deletions are rarely lethal in bacteria such as *E. coli* or *B. subtilis* contrast with the major role that RipA (or its homologs) plays in *Corynebacteriales*, where deletion/depletion of this single enzyme leads to severe defects in cell division and compromises cell viability. The crystal structure of full-length Cg1735, the major PG septal hydrolase required for cell separation in *Cglu*, reveals the enzyme locked in an autoinhibited conformation by the association of the N-terminal coiled-coil domain to the C-terminal catalytic site. The two domains are connected by an extensive linker region, which undergoes a disorder-to-order transition in response to protein–protein interactions, emphasizing the relevance of molecular context on the structure and function of marginally stable coiled-coil domains. The structure strongly suggests an activation mechanism mediated by protein–protein interactions, and we identified Cg1604, a (β/α) protein highly conserved in *Corynebacteriales*, as the protein that directly activates Cg1735. The Cg1604 homolog in *Mtb*, Rv1698 (30% sequence identity), was originally reported by Niederweis and co-workers to function as a channel-forming outer membrane protein involved in copper efflux and renamed MctB (for Mycobacterial copper transport protein B) (29–31). However, the same authors subsequently suggested that Rv1698 was possibly anchored in the inner membrane and could fulfill a more pleiotropic role (32), in line with our model (Fig. 5). The crystal structure of monomeric Cg1604 (Fig. 3*B*) does indeed rule out a putative channel-forming property of the protein. Consistent with a regulatory role for Cg1604 homologs in mycobacterial cell separation, a *M. smegmatis* strain lacking expression of MS3747 exhibited severe growth defects, which were restored by expression of the protein (31), and the overexpression of Rv1698 was thought to facilitate nutrient uptake by introducing changes in the cell wall (32). Moreover, transposon insertion mutants of *Mycobacterium avium* at the genetic locus Maa2520/Maa2521 (the Cg1603/Cg1604 homologs) showed increased Congo red binding, indicative of cell wall modifications, and were rendered susceptible to various antibiotics (33), phenocopying the ethambutol sensitivity of Δcg1603 and Δcg1604 in *Cglu* (17) and supporting a similar functional role of these proteins in cell wall remodeling.

The overall conservation of the Cg1735 activation mechanism (Fig. 5) in *Corynebacteriales* has important implications for cell separation in *Mtb*. Besides interacting with other PG remodeling enzymes such as resuscitation-promoting factors (13) or the penicillin-binding protein PonA1 (11), *Mtb* RipA—the Cg1735 homolog—was proposed to be expressed as a zymogen requiring proteolytic processing. This hypothesis is supported by the detection of truncated RipA species in vivo (9) and the observation that cell division was impaired at acidic pH in an *Mtb* strain deficient for MarP, a protease that can cleave RipA in vitro (34). Moreover, the X-ray structures of truncated forms of *Mtb* RipA revealed that the N-terminus of the protein constructs (corresponding to the linker region between the coiled-coil and the CD in full-length RipA) blocked the catalytic cleft in the crystals (10, 21) and might therefore function as the zymogen prodomain. These observations suggest a proteolytic activation mechanism in which the N-terminal coiled-coil domain of RipA would play no major role (35, 36). One difficulty with an activation mechanism based only on proteolysis is that a highly labile RipA linker region (as suggested by the structure of full-length Cg1735) could be prone to non-specific processing, which is indeed consistent with the smear of in vivo processed forms of RipA in Western blotting (9), and therefore to uncontrolled PG hydrolysis.

Our structural and biochemical results now shed new light on these data, highlighting a more complex RipA activation mechanism in which both proteolysis and protein–protein interactions can play a role. Indeed, the predicted structure of the *Cglu* Cg1735–Cg1604 activation complex (Fig. 3*E*) is identical to those for the putative homologous complexes from *Mtb* (RipA-Rv1698) or *M. smegmatis* (RipA-MS3747) (*SI Appendix,* Fig. S12*A*), and the molecular interfaces involved in these complexes are also highly conserved among mycobacterial RipA and Rv1698 homologs (*SI Appendix,* Fig. S12*B*), lending strong support to the presence of a similar activation complex and regulation mechanism in *Mtb*, mediated by the Cg1603/Cg1604 homologs Rv1697/Rv1698. Such a mechanism puts RipA under direct control of the divisome and might explain why cell division is normal in MarP-deficient *Mtb* cells at neutral pH (34). Furthermore, a weaker intramolecular association at acidic pH between the coiled-coil and CDs of RipA (as seen for Cg1735, Fig. 4*D*) might also explain why RipA is activated by proteolysis under these conditions but not at neutral pH (34), where a stronger intramolecular CC1-CD association would keep the catalytic cleft bound to the coiled-coil domain, even upon cleavage of the connecting region. Our coiled-coil-mediated autoinhibition model of RipA challenges a previous hypothesis proposing that a loop within the linker region would fulfill such an inhibitory role (10, 36) and explains why removal of this loop did not change the RipA activity on high-molecular weight PG (21).

In many bacteria, septal hydrolysis requires FtsE and FtsX (24), two proteins that form an ABC-transporter-like complex (FtsEX), originally implicated in the control of cell wall hydrolase activity in many bacteria across various phyla (20, 37–39). The functional conservation and wide distribution of *ftsEX* genes in bacterial genomes suggest a fundamental role of the complex in signal transduction coupling PG hydrolysis to cell division. A currently accepted model is that, upon signal sensing, ATP hydrolysis by FtsE promotes a conformational change transmitted across the membrane via FtsX, causing the extracellular domain of FtsX to interact with either cell wall hydrolases or effector proteins to activate PG hydrolysis (25, 40, 41). The *ftsEX* genes are also present in *Corynebacteriales*, although little is known about their function. In *Mtb*, FtsX was shown to activate RipC, another NlpC/P60 hydrolase with an N-terminal coiled-coil domain that belongs to a different clade than RipA (Fig. 1*A*). Unlike RipA, however, both FtsX and RipC are non-essential proteins (42) dispensable for cell division (20, 43), suggesting the presence of two distinct regulation mechanisms of PG hydrolysis. In agreement with this hypothesis, *Mtb* FtsX was found to interact with RipC, but not with RipA (20). It was therefore intriguing that, in *Cglu*, FtsX was reported (17) to form a complex with the RipA homologue Cg1735 (mistakenly referred to as RipC in that work) using the POLAR two-hybrid approach (44). However, the authors described aggregation issues of Cg1735 when expressed in *E. coli*, which are consistent with the large unstructured region of the protein revealed by our crystallographic study and might account for non-specific binding in the POLAR assay. When we modeled a full-length FtsX dimer in complex with Cg2401 (the homolog of *Mtb* RipC) using AlphaFold, the structure showed the periplasmic domain of FtsX bound to the tip of the conserved coiled-coil domain from the hydrolase with a 2:1 stoichiometry (*SI Appendix,* Fig. S13*A*), as seen for the crystal structure of *E. coli* EnvC in complex with the FtsX periplasmic domain (41). The predicted interface on the tip of the Cg2401 coiled-coil domain is highly conserved in the whole RipC family from *Corynebacteriales* (*SI Appendix,* Fig. S13*B*), suggesting that the FtsEX complex regulates Cg2401 in *Cglu* in a similar way as it regulates RipC in *Mtb*. Thus, based on our and previous results, we propose that *Corynebacteriales* have evolved at least two distinct regulatory systems based on protein–protein interactions to ensure divisome control of cell wall hydrolysis, a first one involving the Cg1603/Cg1604-mediated regulation of RipA (Cg1735), which plays a primary role in cell separation, and a second one involving the FtsEX-mediated control of RipC (Cg2401), whose physiological role remains poorly understood.

## Material and Methods

### Phylogenetic Analyses.

We assembled a database of 51 proteomes representing all *Corynebacteriales* diversity present at the GenBank database (45) as of August 2021. We selected five species per genus—if available—and we chose preferably the most complete assemblies (for a list of taxa see *SI Appendix,* Table S1). We used HMM profile searches to identify all members of the NlpC/P60 superfamily in the database. First, we used the HMMER package (v3.3.2) (46) tool jackhmmer to look for homologs of *Mtb* RipA in all the proteomes using the GenBank sequence BAB98931.1 as query. The hits were aligned with mafft (v7.475) (47) using default parameters. The alignments were manually curated, removing sequences that did not contain the NlpC/P60 domain, and trimmed, keeping only the NlpC/P60 domain. The hits obtained by jackhmmer might not include sequences that are very divergent from the single sequence query. For this reason, the trimmed alignment was used to create an HMM profile using the HMMER package (v3.3.2) tool hmmbuild. This specific and curated HMM profile for the NlpC/P60 domain was used for a second and final round of searches against the proteomes using the HMMER tool hmmsearch. The new hits were aligned with linsi, the accurate option of mafft (v7.475), and trimmed using trimAl (v1.2) (48) to keep only the positions with less than 50% of gaps (–gt 0.5). NCBI GenBank accession numbers of the sequences used in the phylogenetic analysis are provided in *SI Appendix,* Table S1, and the alignment and phylogeny file are provided in *SI Appendix*. The trimmed alignment was used to reconstruct the phylogeny of the NlpC/P60 superfamily. We used the maximum-likelihood phylogeny reconstruction tool IQ-TREE (v2.0.6) (49), with the LG + F + R7 model (–m MFP) and ultrafast bootstraps (–B 1000). For each NlpC/P60 sequence, we retrieved from UniProt (50) the position of annotated signal peptides, coiled-coil domains, and NlpC/P60 domains. This information was mapped on the *Corynebacteriales* NlpC/P60 superfamily phylogeny using the online tool iTOL (51) and custom scripts.

### Bacterial Strains and Growth Conditions.

*Escherichia coli* DH5α or CopyCutter EPI400 were used for cloning and were grown in Luria-Bertani (LB) broth or agar plates at 37 °C supplemented with 50 µg/mL kanamycin or 100 µg/mL carbenicillin when required. For soluble protein production, *E. coli* BL21 (DE3) was grown in 2YT broth supplemented with 50 µg/mL kanamycin or 100 µg/mL carbenicillin at the appropriate temperature.

### Cloning for Recombinant Protein Production in E. Coli.

All plasmids used in this study are listed in the *SI Appendix,* Table S3. Fragments of *Cglu* ATCC13032 genetic material encoding the full periplasmic region of cg1735 (Cg1735 construct, residues 20 to 600), the two coiled-coil domains (Cg1735_ΔCD_ construct, residues 20 to 386), and the globular domain of cg1604 (Cg1604 construct, residues 36 to 295) were cloned by Gibson Assembly into a pET15-derivative vector containing an N-terminal 6×His-SUMO tag using primers described in *SI Appendix,* Table S4. The plasmids for expression of the CD of cg1735 (Cg1735_CD_ construct, residues 460 to 600) and the CC1 domain (Cg1735_CC1_ construct, residues 20 to 238) were obtained by gene deletion using pUMS_216 and pUMS_217, respectively, as templates. The plasmids for expression of the point mutants Cg1604_L146R_ and Cg1735_E69K, N72D_ were obtained by site-directed mutagenesis. All plasmids were verified by Sanger sequencing (Eurofins Genomics).

### Soluble Protein Expression and Purification.

N-terminal 6×His-SUMO-tagged constructs were expressed in *E. coli* BL21 (DE3) following an autoinduction protocol. After 4 h at 37 °C, cells were grown for 20 h at 18 °C in 2YT complemented autoinduction medium (52) containing 100 µg/mL carbenicillin. Cells were harvested and flash frozen in liquid nitrogen. Cell pellets were resuspended in lysis buffer (50 mM Hepes pH 7.5, 300 mM NaCl, 5 mM imidazole, 5% glycerol, 1 mM MgCl_2_, benzonase, lysozyme, 0.25 mM Tris (2-carboxyethyl) phosphine hydrochloride (TCEP), EDTA-free protease inhibitor cocktails (Roche)) at 4 °C and lysed by sonication. The lysate was centrifuged (15,000 g) for 15 min at 4 °C, and the supernatant was loaded onto a Ni-NTA affinity chromatography column (HisTrap FF crude, Cytiva) pre-equilibrated in buffer A (50 mM Hepes pH 7.5, 300 mM NaCl, 10 mM imidazole, 5% glycerol). His-tagged proteins were eluted with a linear gradient of buffer B (50 mM Hepes pH 7.5, 300 mM NaCl, 1 M imidazole, 5% glycerol). The fractions of interest were pooled and dialyzed in the presence of the SUMO protease at a 1:100 w/w ratio. Dialysis was carried out at 4 °C overnight in SEC buffer (25 mM Hepes pH 7.5, 150 mM NaCl, 5% glycerol). Cleaved His-tags and His-tagged SUMO protease were removed with Ni-NTA agarose resin. The cleaved protein was concentrated and loaded onto a Superdex 75 or 200 16/60 size exclusion column (GE Healthcare) pre-equilibrated at 4 °C in SEC buffer. The peak corresponding to the protein was concentrated, flash frozen in small aliquots in liquid nitrogen, and stored at −80 °C. Purity was verified by sodium dodecyl sulfate–polyacrylamide gel electrophoresis (SDS-PAGE).

### Selenomethionine-Derived Protein Expression and Purification.

Se-Met-derived Cg1604 was expressed in *E. coli* BL21 (DE3) with all media containing 50 µg/mL carbenicillin. Cells were grown for 8 h at 37 °C in 2YT medium and inoculated 1:100 in M9 medium (33.7 mM Na_2_HPO_4_-2H_2_O, 22.0 mM KH_2_PO_4_, 8.6 mM NaCl, 9.4 mM NH_4_Cl, 2 mM MgSO_4_, 0.3 mM CaCl_2_ 0.4% (w/v) D-glucose, 3.8 µM thiamin, 4.1 µM biotin). The next day, the overnight culture was diluted 1:50 in fresh M9 medium and grown until OD600 = 0.6. The methionine biosynthetic pathway was inhibited by adding lysine, phenylalanine, and threonine at 100 mg/L; isoleucine and valine at 50 mg/L; and selenomethionine at 60 mg/L. Protein expression was induced 30 min after addition of amino acids by adding IPTG to a final concentration of 1 mM, and cells were grown for 20 h at 18 °C, harvested, and flash frozen in liquid nitrogen. Protein purification was performed as described above.

### Crystallization.

Initial screening of crystallization conditions was carried out by the vapor diffusion method using a MosquitoTM nanoliter-dispensing system (*TTP Labtech, Melbourn, United Kingdom*) following established protocols (53). The best crystals of selenomethionine-derived Cg1604 (35 mg/mL) were obtained after 22 d in 30% (w/v) PEG 4 K, 0.2 M MgCl_2_, 0.1 M TRIS pH 8.5. Optimal trigonal crystals of Cg1735 (15.4 mg/mL) were obtained after 7 d in 1 M (NH_4_)_2_H Citrate, 0.1 M Na Acetate pH 4.6. For phasing, crystals were soaked in 10 mM Cl_4_K_2_Pt for 5 min. Orthorhombic crystals of Cg1735 (15.4 mg/mL) were obtained after 63 d in 2.4 M (NH_4_)_2_SO_4_, 0.1 M Na_3_ citrate. All crystals were cryo-protected in mother liquor supplemented by 33% (vol/vol) glycerol or ethylene glycol.

### Data Collection, Structure Determination, and Refinement.

X-ray diffraction data were collected at 100 K at the Synchrotron Soleil (Saclay, France). All datasets were processed using XDS (54) and AIMLESS from the CCP4 suite (55) (*SI Appendix,* Table S2). Merged data from Cg1735 crystals were further subjected to anisotropy correction with STARANISO (56), the elliptical diffraction limits were d_h00_ = d_0k0_ = 4.8 Å, d_00l_ = 2.9 Å for the trigonal dataset and d_h00_ = 4.5 Å, d_0k0_ = 6.0 Å, d_00l_ = 4.0 Å for the orthorhombic dataset. The structure of trigonal Cg1735 soaked in Cl_4_K_2_Pt was determined by SAD phasing: two major and several minor Pt sites in the asymmetric unit were found with SHELXD (57), and the experimental phases obtained with PHASER (58) were significantly improved by density modification with PARROT from the CCP4 suite, as the crystal has a high (85%) solvent content. Coiled-coils were clearly visible in the resulting electron density map (*SI Appendix,* Fig. S6), and the CD was positioned by real-space molecular replacement using *Mtb* RipB (PDB code 3pbi) as search model. The structure of the orthorhombic form of Cg1735 was solved by molecular replacement methods, and that of SeMet-labeled Cg1604 by SAD phasing using SHELXD and automatic model building with BUCCANEER from the CCP4 suite. All structures were refined through iterative cycles of manual model building with COOT (59) and reciprocal space refinement with PHENIX (60) or BUSTER (61). Non-crystallographic symmetry and secondary structure restraints were applied to both Cg1735 structures and, for the lower resolution orthorhombic structure, additional homology model (*Mtb* RipB) torsion restraints were applied to the CD. The final refinement statistics are shown in *SI Appendix,* Table S2 and representative views of the final electron density map for each structure are shown in *SI Appendix,* Fig. S14. Structural figures were generated with ChimeraX (62).

### AlphaFold Prediction of Complex Structures.

The predicted structures of the different complexes were obtained with AlphaFold-Multimer by submitting the different sequences to the server at: https://colab.research.google.com/github/sokrypton/ColabFold/blob/main/AlphaFold2.ipynb (63). The structures shown correspond to those ranked first according to model confidence as described (64). The ipTM values for the different complexes are the following: 0.75 (Fig. 3*E*); 0.72 and 0.70 (for the *Mtb* and *M. smegmatis* complexes respectively, *SI Appendix,* Fig. S12*A*); and 0.64 (*SI Appendix,* Fig. S13*A*).

### RipA Endopeptidase Activity Tests.

*B. subtilis* PG extract was purchased from Sigma and labeled with Remozol Brilliant Blue (RBB) (Sigma) as described (20). The activity assays were performed in activity buffer (25 mM succinate pH 5.0, 150 mM NaCl, 5% glycerol, 0.25 mM TCEP) using 25 µM Cg1735 in the presence or absence of 50 µM Cg1604 (Fig. 3*A*) or 50 µM Cg1735_CD_ in the presence or absence of 50 µM Cg1735_CC1_ (Fig. 2*D*). The reaction mixtures (100 µL, 0.1 mg PG-RBB) were incubated at 20 °C for 48 h, the reactions stopped by centrifugation at 18,000 × g for 15 min at 4 °C, the supernatants transferred to 1 cm quartz cell, and the absorbance measured at 595 nm. Reactions were performed in triplicate. The activities of buffer alone and lysozyme (50 µM) were used as negative and positive controls, respectively.

### Circular Dichroism.

Far-UV CD spectra (185 to 260 nm) were recorded with a Jasco J-1500 spectropolarimeter (Jasco, Tokyo, Japan) with a 450 W lamp at 20 °C using 25 mM HEPES pH 7.5 150 mM NaCl 5% glycerol and a 50-µm-path-length quartz Suprasil cell (Starna), with protein concentrations between 1.9 and 3.2 mg/mL. (+)-camphor-10-sulfonic acid (CSA) was used to calibrate amplitudes and wavelength positions of the CD experiment. Spectral acquisitions of 0.1 nm steps at 1 s integration time, with a bandwidth of 1 nm, were performed four times for the samples as well as for the buffer. The measurements were carried out with constant nitrogen gas flux of 10 mL/min.

Acquisitions were averaged, and buffer baseline was subtracted with SpectraManager (JASCO). No smoothing was applied. CDtoolX (65) was used to zero between 255 to 260 nm and to calibrate the signal amplitude from the fresh CSA signal. Data are presented as delta epsilon (Δε) per residue (L.mol-1.cm-1.residue-1) calculated using the molar concentration of protein and number of residues.

### Analytical Ultracentrifugation.

Cg1735 was diluted to 5 mg/mL in 25 mM HEPES, pH 7.5, 150 mM NaCl, 5% glycerol, and centrifuged at 42,000 rpm in an Optima analytical ultracentrifuge (Beckman Coulter) at 20 °C in an eight-hole AN 50–Ti rotor equipped with 3-mm and 12-mm double-sector aluminum epoxy centerpieces. Detection of the biomolecule concentration as a function of radial position and time was performed by absorbance measurements at 280 nm and by interference detection. Sedimentation velocity data analysis was performed by continuous size distribution analysis c(s) using Sedfit software. All the c(s) distributions were calculated with a fitted fractional ratio f/f0 and a maximum entropy regularization procedure with a confidence level of 0.95. Partial specific volume, buffer viscosity, and density were calculated using the Sednterp software.

### Analytical SEC.

To obtain the chromatograms shown in Fig. 4, protein samples were diluted in SEC buffer (25 mM Hepes, pH 7.4, 150 mM NaCl, 5% glycerol; or 25 mM Succinate, pH 5.0, 150 mM NaCl, 5% glycerol). Protein mixtures were incubated in an equimolar ratio (either 50 µM or 80 µM) on ice for 1 h, centrifuged, and eluted on a Superdex 200 Increase 5/150 GL (Cytivia) pre-equilibrated with SEC buffer at 4 °C. Fractions of 100 µL were collected for analysis by SDS-PAGE.

### BLI Assays.

The affinity of Cg1735_CD_ for His_SUMO_Cg1735_CC1_, and that of Cg1735_CD_ and Cg1604 for biotinylated Cg1735_ΔCD_ were measured in real time using a BLI Octet-Red384 device (Pall ForteBio) at 25 °C.

His_SUMO_Cg1735_CC1_ was diluted at 3 μg/mL in buffer A (HEPES 25 mM pH 7.5, NaCl 150 mM, glycerol 5%) supplemented with BSA 1 mg/mL and immobilized on the commercially available Sartorius Ni-NTA biosensors for 10 min at 1,000 rpm followed by a washing step in the same buffer for 3 min to remove any loosely bound protein. Empty sensors were used as reference for unspecific binding. His-SUMO-Cg1735_CC1_ loaded or empty reference sensors were incubated for 5 min at 1,000 rpm in the absence and presence of twofold serially diluted concentrations of Cg1735_CD_ (20 to 0.3125 μM range) in buffer A supplemented with BSA at 1 mg/mL.

For the biotinylation reaction, 100 μL of recombinant Cg1735_ΔCD_ (25 μM) was incubated with 20 molar excess of EZ-Link NHS-PEG4-Biotin (Thermo Scientific) following supplier instructions. Biotinylated Cg1735_ΔCD_ was diluted at 10 μg/mL in buffer A (HEPES 25 mM pH 7.5, NaCl 150 mM, glycerol 5%) and immobilized on the commercially available Sartorius Streptavidin biosensors for 5 min at 1,000 rpm followed by a washing step in buffer A for 3 min to remove any loosely bound protein. Empty sensors were used as reference for unspecific binding. Cg1735_ΔCD_-loaded or empty reference sensors were incubated for 5 min at 1,000 rpm in the absence and presence of twofold serially diluted concentrations of Cg1735_CD_ (20 to 0.3125 μM range) or Cg1604 (200 to 3.125 μM range) in buffer A supplemented with BSA at 1 mg/mL.

Specific signals were obtained by subtracting both non-specific signals measured on empty sensors and buffer signals on specific His-SUMO-Cg1735_CC1_ or biotinylated Cg1735_ΔCD_-loaded sensors. Assays were performed at least twice, and *Kd* values were obtained from steady-state signal versus concentration curves fitted with GraphPad Prism 9 assuming a one-site binding model.

## Supplementary Material

Appendix 01 (PDF)

## Data Availability

Atomic coordinates and structure factors have been deposited in the Protein Data Bank with accession codes 8AUC, 8AUD, and 8AU6 (*SI Appendix,* Table S2). All data used to produce the phylogenetic analysis is provided as Supporting Data in the Mendeley Data repository (https://data.mendeley.com/datasets/2543ntd7pt/draft?a=6249d3ed-c5c2-404c-8049-c041ec488152).
