# Supplementary material for: FtsEX-independent control of RipA-mediated cell separation in Corynebacteriales
Source: Proc Natl Acad Sci U S A. 2022 Dec 5;119(50):e2214599119. doi: 10.1073/pnas.2214599119 (PMC9897464; doi:10.1073/pnas.2214599119)
Supplement: Supplementary file 1 — Appendix 01 (PDF) [file pnas.2214599119.sapp.pdf]

PNAS

Supporting Information for

FtsEX-independent control of RipA-mediated cell separation in *Corynebacteriales*.

Quentin Gaday, Daniela Megrian, Giacomo Carloni, Mariano Martinez, Bohdana Sokolova,

Mathilde Ben Assaya, Pierre Legrand, Sebastien Brûlé, Ahmed Haouz,

Anne Marie Wehenkel, and Pedro M Alzari

Correspondence to A.M. Wehenkel and P.M. Alzari

Email: anne-marie.wehenkel@pasteur.fr and pedro.alzari@pasteur.fr

This PDF file includes:

Figures S1 to S14

Tables S1 to S4

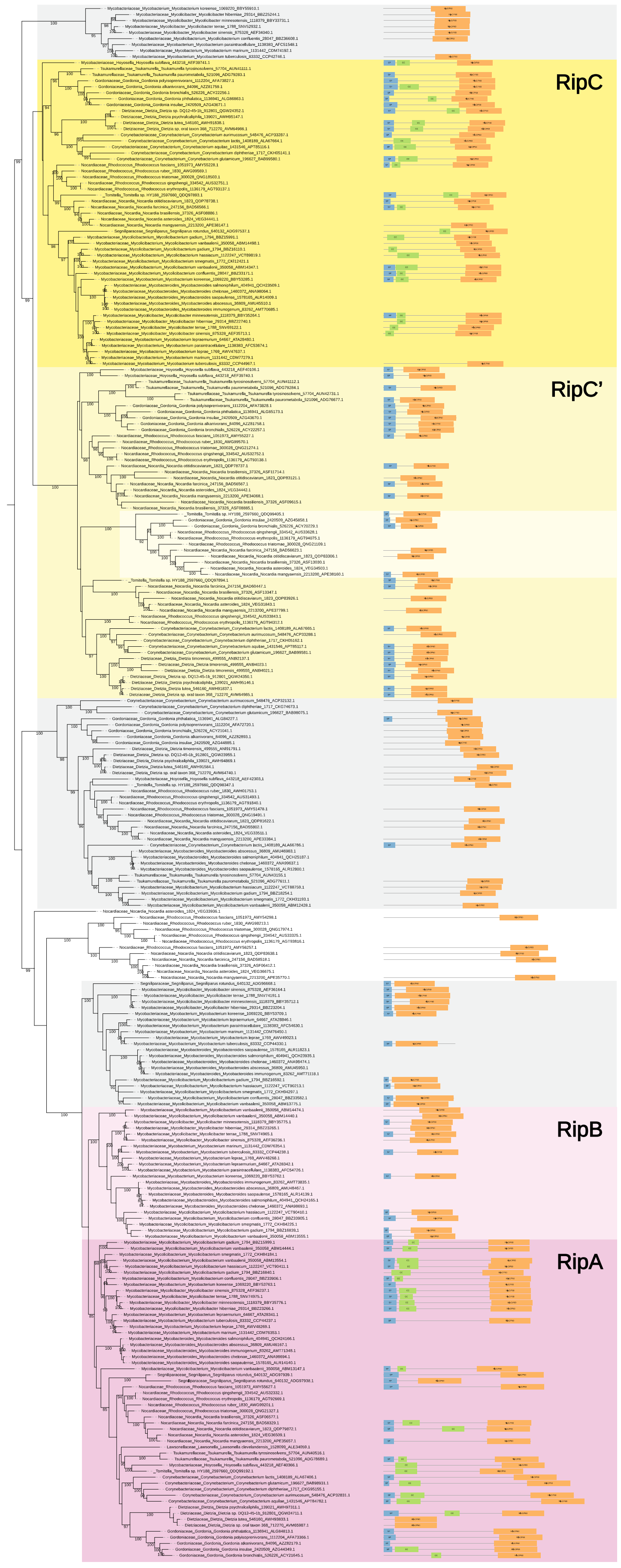

Fig. S1. Phylogeny of the NlpC/P60 superfamily in *Corynebacteriales* (full version of Fig. 1A)

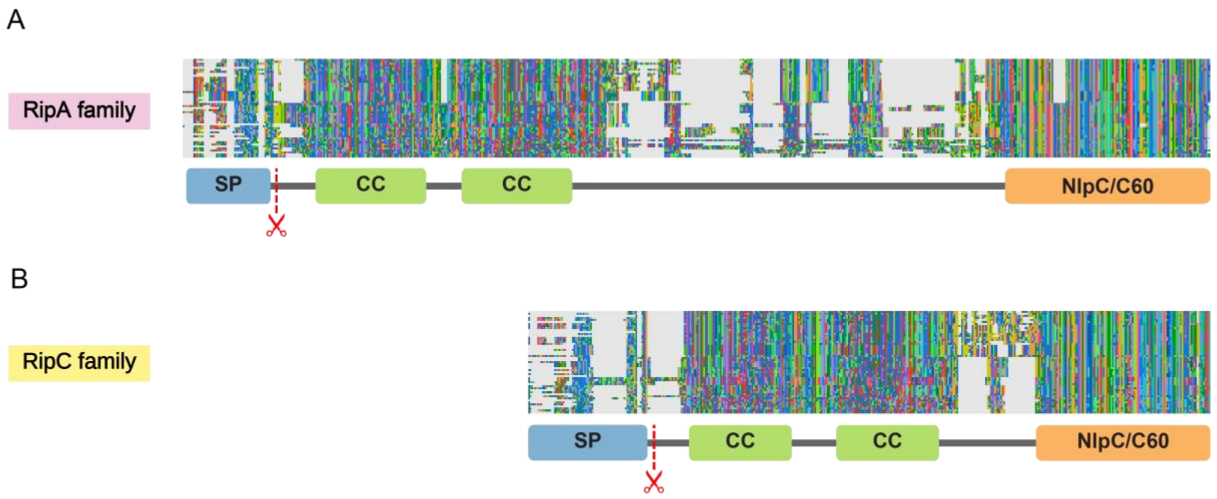

Fig. S2. Multiple alignment of two families of NlpC/P60 PG hydrolases: (A) the RipA/Cg1735 family, and (B) the RipC/Cg2401 family.

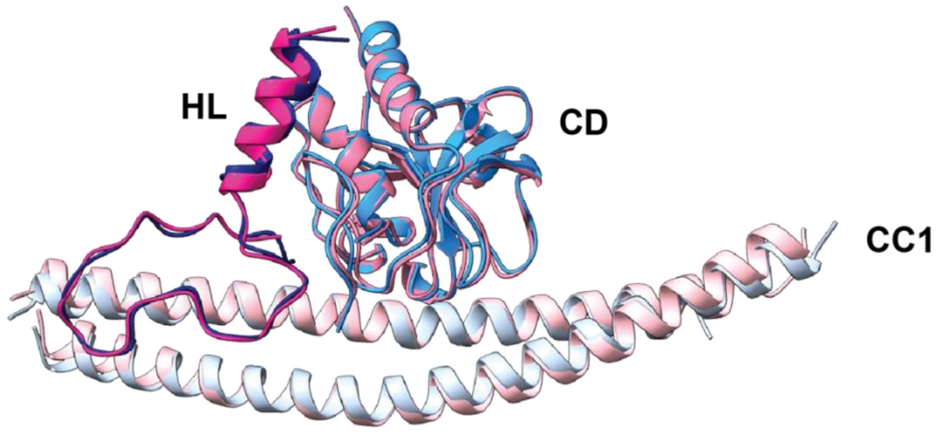

Fig. S3. Overall superposition of the autoinhibited RipA structures from the orthorhombic crystal form (red) and the equivalent region from the trigonal crystal form (blue). The 3 structural domains (CC1, HL and CD) are indicated. In the trigonal model, the CC1 domain shown corresponds to the second monomer in the asymmetric unit (see Figure S5).

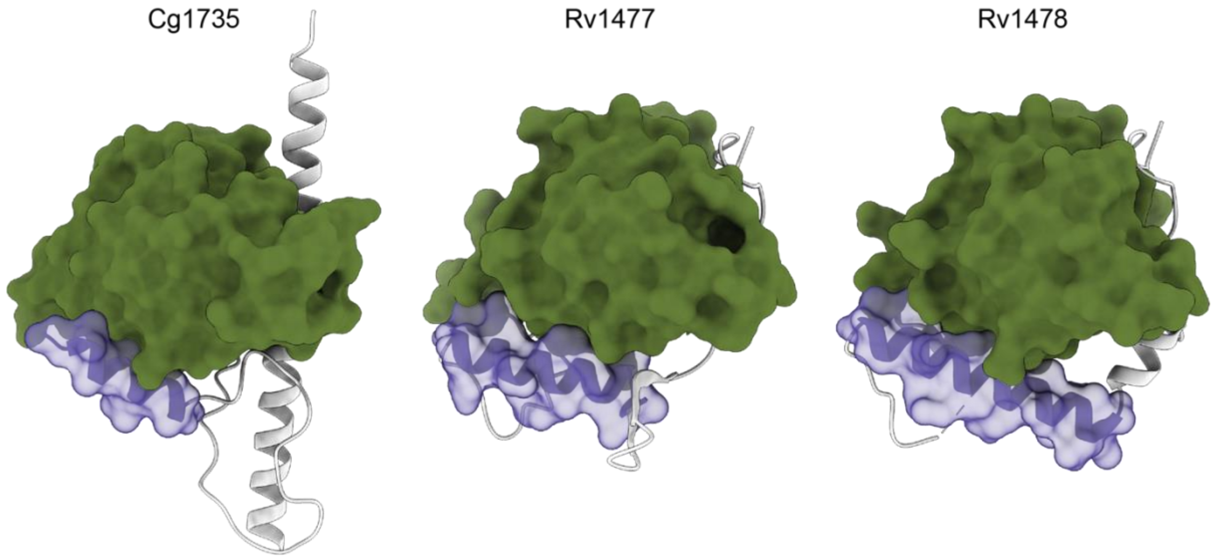

Fig. S4. The NplC/P60 catalytic cores (in green) of *Cglu* Cg1735 (this work), *Mtb* RipA (PDB code 3pbc) and *Mtb* RipB (PDB code 3pbi) are shown in a similar orientation. In all three structures, the NlpC/P60 domain binds an additional α-helix (in blue) at an equivalent position.

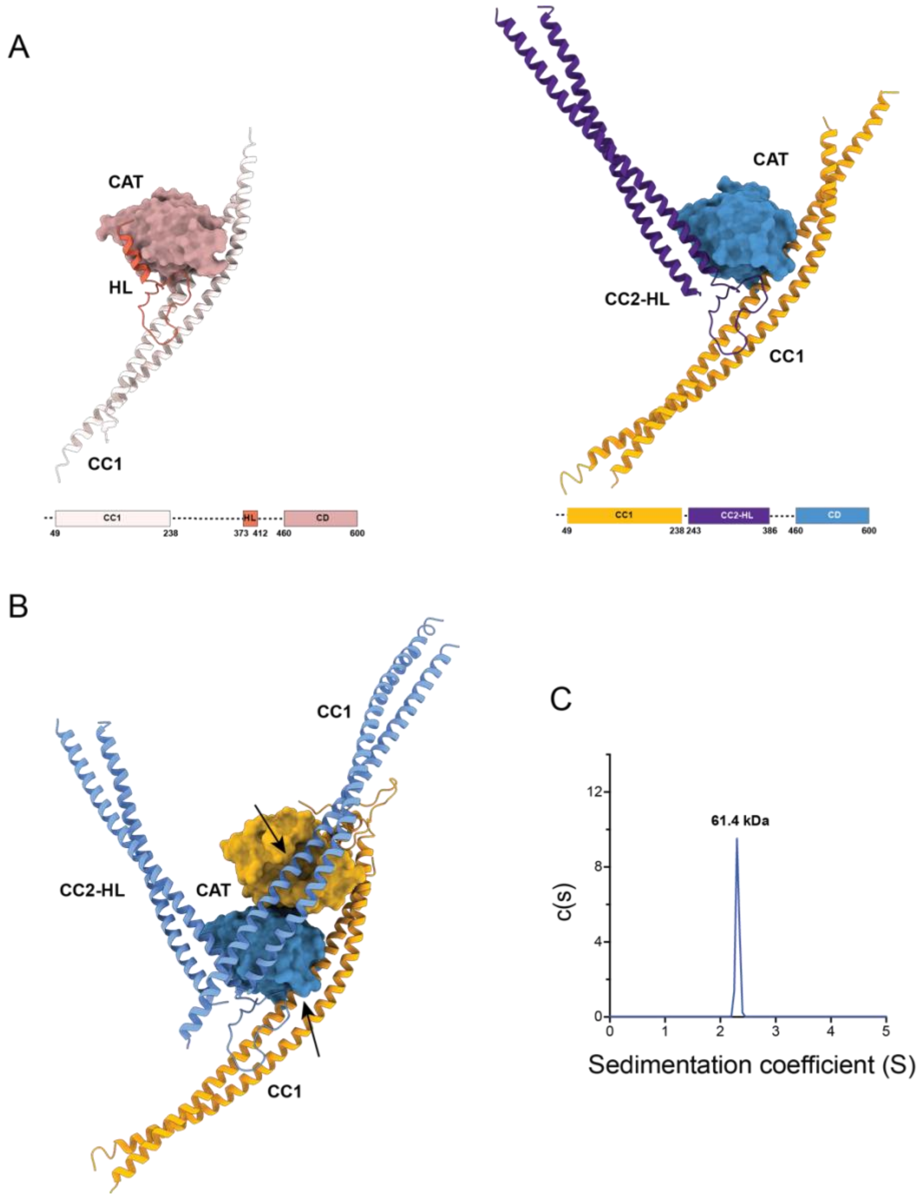

Fig. S5. Structural comparison of the two crystal forms of Cg1735. (A) The short α-helix (orange) packed against the catalytic domain (pink surface) in the orthorhombic form (left) extends into a two-helical coiled-coil domain, CC2, in the trigonal form (right). The CC1 domain (yellow) corresponds to the second molecule in the trigonal asymmetric unit. (B) Structure of the full Cg1735 dimer in the trigonal form, with one monomer oriented as in (A) and color-coded by polypeptide chain (the second CC2-HL is hidden by the catalytic domain in this view). Individual structural domains (CC1, CC2-HL, CAT) are labelled, and arrows indicate the active sites. (C) Sedimentation coefficient distribution of Cg1735 showing a single peak for the monomeric form of Cg1735 at the highest tested concentration (5 mg/ml).

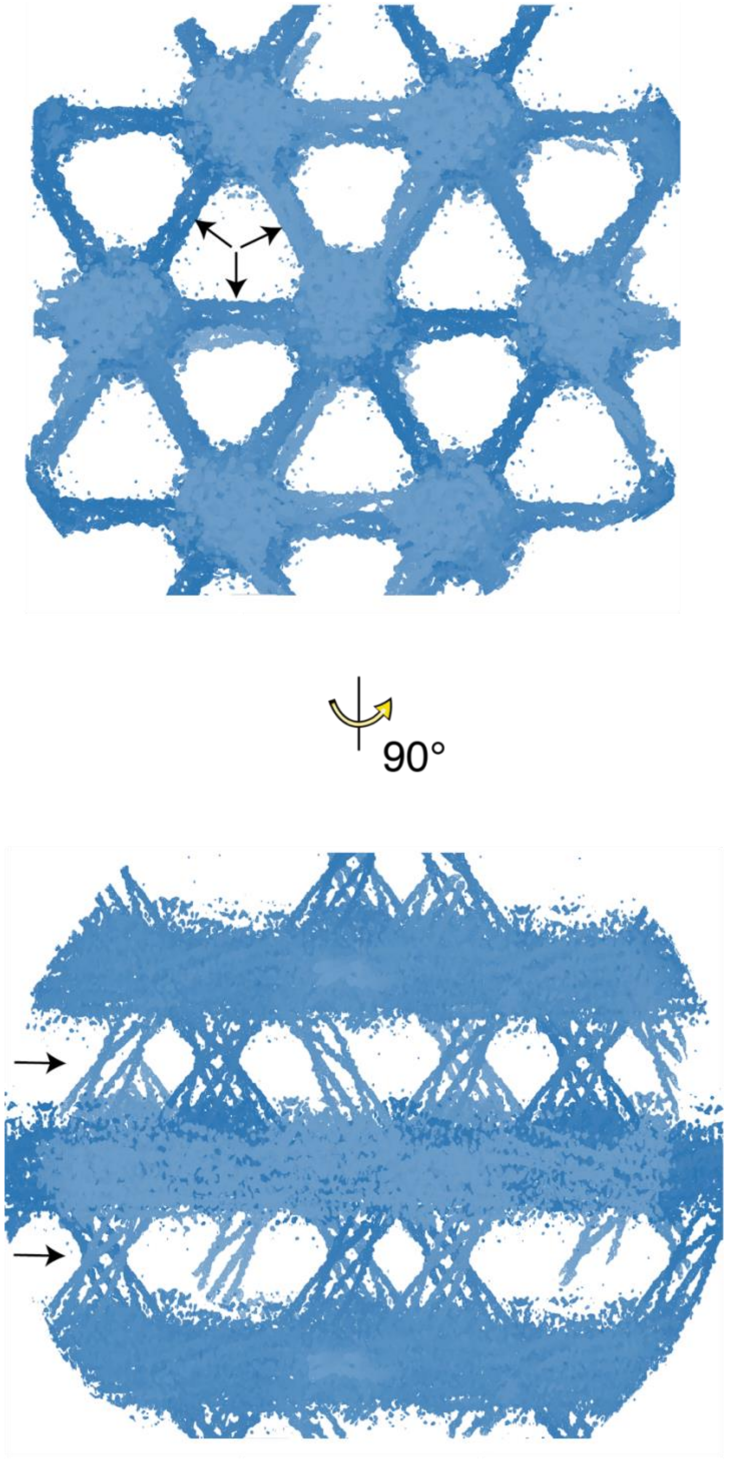

Fig. S6. Projections perpendicular (top view) and parallel (bottom view) to the crystallographic ***c*** axis of the SAD-phased electron density map for the trigonal crystal form (85% of solvent content). The coiled-coil CC2 domains (indicated by arrows) are engaged in extensive crystal packing interactions.

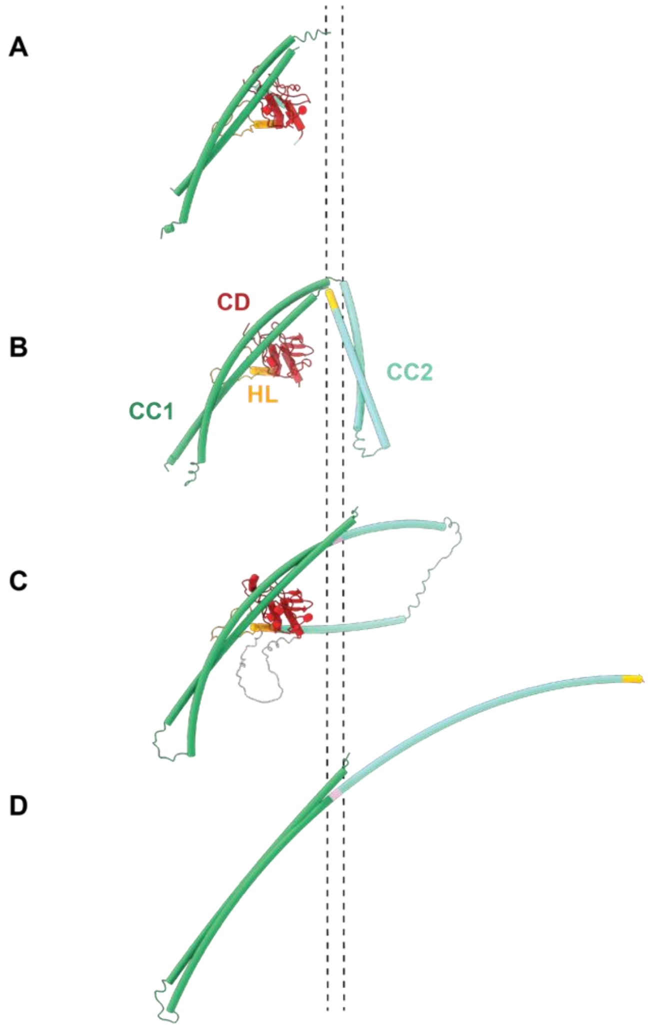

Fig. S7. Our crystallographic and AlphaFold2 (AF2)-predicted structures of Cg1735 suggest that, depending on the molecular context, the coiled-coil helices may adopt different structures. (a) The orthorhombic crystal structure revealed the CC1 domain (green) bound to the catalytic domain (red); (b) the trigonal crystal structure showed the formation of a second coiled-coil, CC2 (cyan) (the catalytic domain shown here comes from the second molecule in the asymmetric unit); (c) the AF2 model of full-length Cg1735 suggests a single α-helical extension of CC1, and breaks down the CC2 domain to preserve the interaction of the catalytic domain with the HL and CC1 domains; and (d) the AF2 model of Cg1735 without the catalytic domain (Cg1735$_{\Delta CD}$) suggest an even longer α-helical extension of CC1.

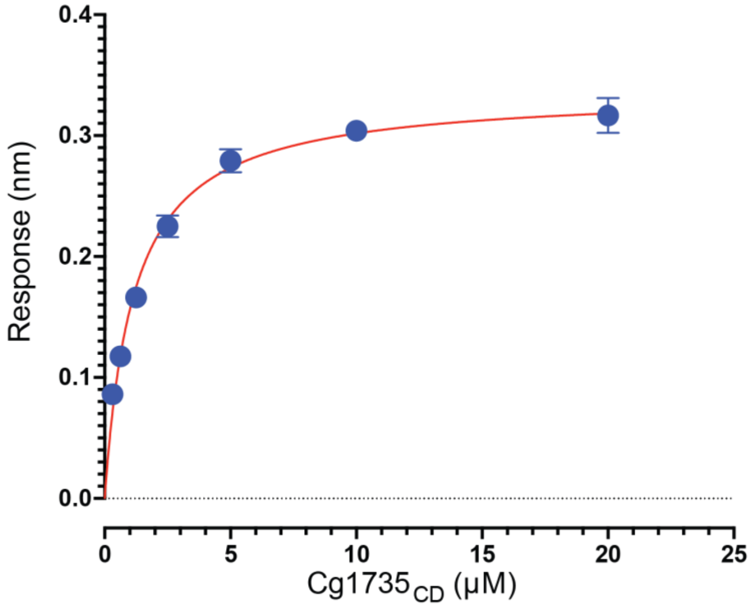

One site binding (hyperbola)	
Best-fit values	
Bmax	0.3366
Kd	1.157
95% CI (profile likelihood)	
Bmax	0.3234 to 0.3505
Kd	0.9895 to 1.349
Goodness of Fit	
Degrees of Freedom	12
R squared	0.9878
Sum of Squares	0.001248
Sy.x	0.01020
Constraints	
Bmax	Bmax > 0
Kd	Kd > 0
Number of points	
# of X values	14
# Y values analyzed	14

Fig. S8. BLI data analysis for Cg1735$_{CC1}$-Cg1735$_{CD}$ interaction. To obtain the *Kd* value (1.2 μM) from the interaction profiles shown in Figure 2B, steady-state signal versus concentration curves were fitted assuming a one site binding model.

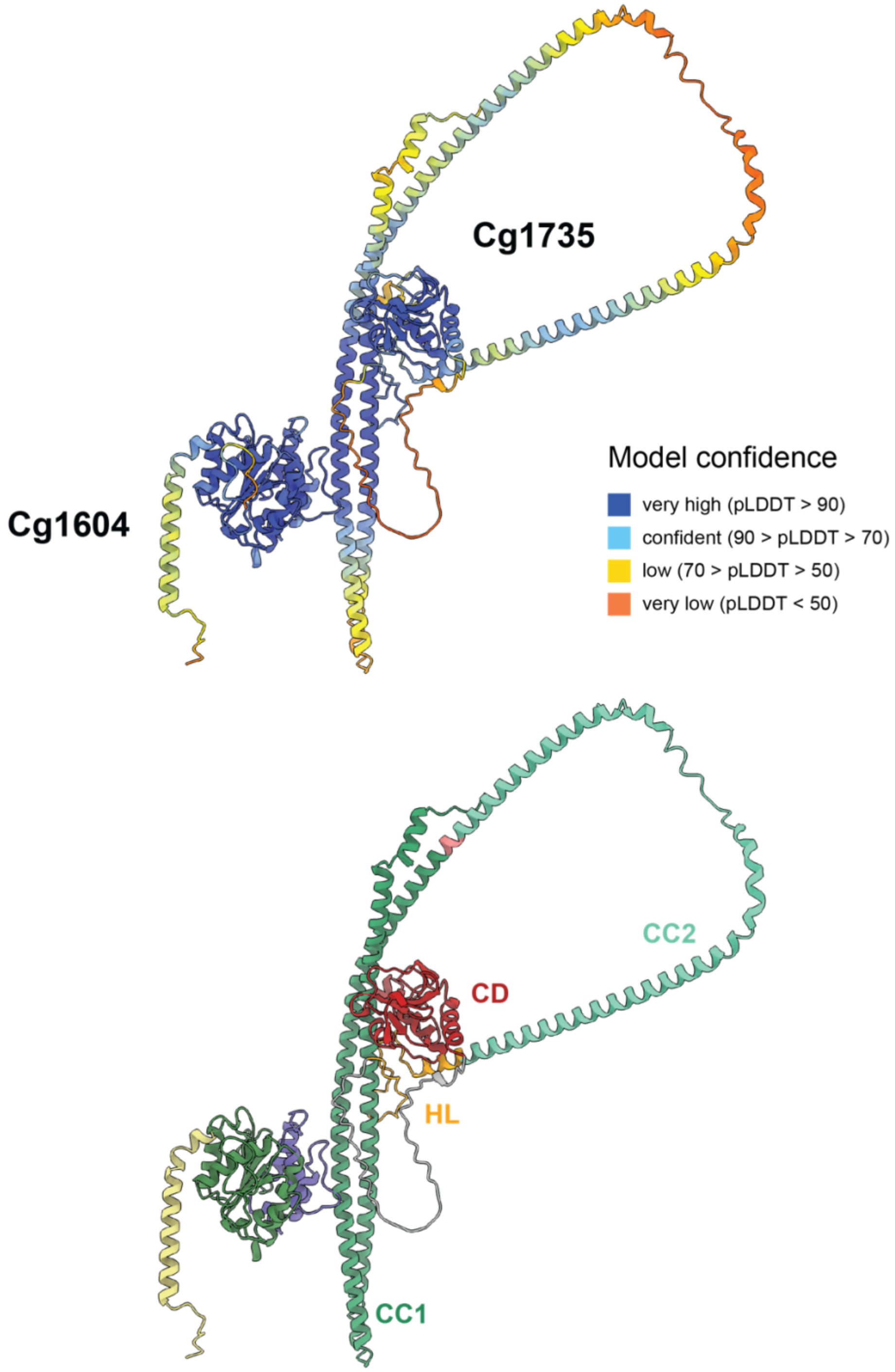

Fig. S9. Predicted AlphaFold2 structure of the Cg1604-Cg1735 complex color-coded by model confidence (top) and domain organization (bottom).

A

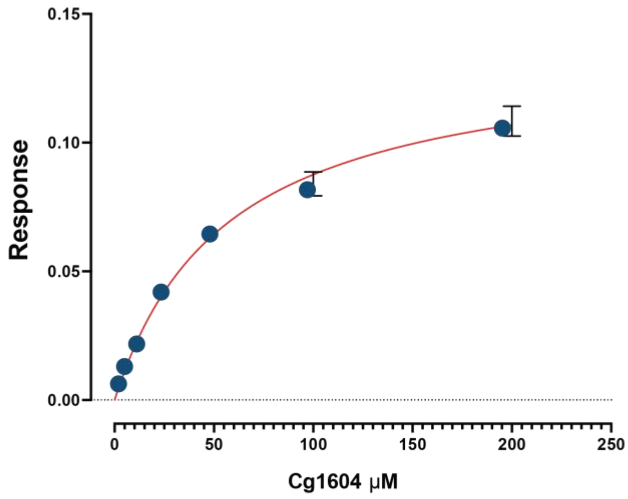

One site binding (hyperbola)	
Best-fit values	
Bmax	0.1369
Kd	56.32
95% CI (profile likelihood)	
Bmax	0.1278 to 0.1473
Kd	47.28 to 67.35
Goodness of Fit	
Degrees of Freedom	12
R squared	0.9934
Sum of Squares	0.0001155
Sy.x	0.003102
Constraints	
Bmax	Bmax > 0
Kd	Kd > 0
Number of points	
# of X values	14
# Y values analyzed	14

B

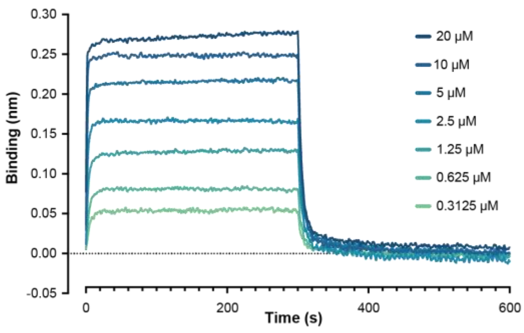

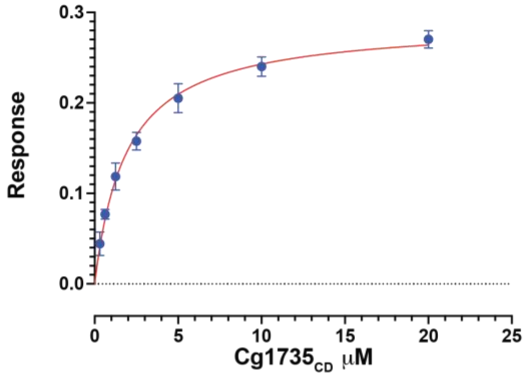

One site binding (hyperbola)	
Best-fit values	
Bmax	0.2889
Kd	1.896
95% CI (profile likelihood)	
Bmax	0.2724 to 0.3067
Kd	1.549 to 2.319
Goodness of Fit	
Degrees of Freedom	12
R squared	0.9848
Sum of Squares	0.001308
Sy.x	0.01044
Constraints	
Bmax	Bmax > 0
Kd	Kd > 0
Number of points	
# of X values	14
# Y values analyzed	14

Fig. S10. BLI data analysis for the interactions of Cg1735$_{\Delta CD}$ with Cg1604. (A) To obtain the *Kd* value (56 µM) for the Cg1735$_{\Delta CD}$-Cg1604 complex from the interaction profiles shown in Figure 4B, steady-state signal versus concentration curves were fitted assuming a one site binding model. (B) For comparison purposes, Cg1735$_{\Delta CD}$ binds the catalytic domain Cg1735$_{CD}$ under the same experimental conditions with an apparent *Kd* value of 1.9 µM.

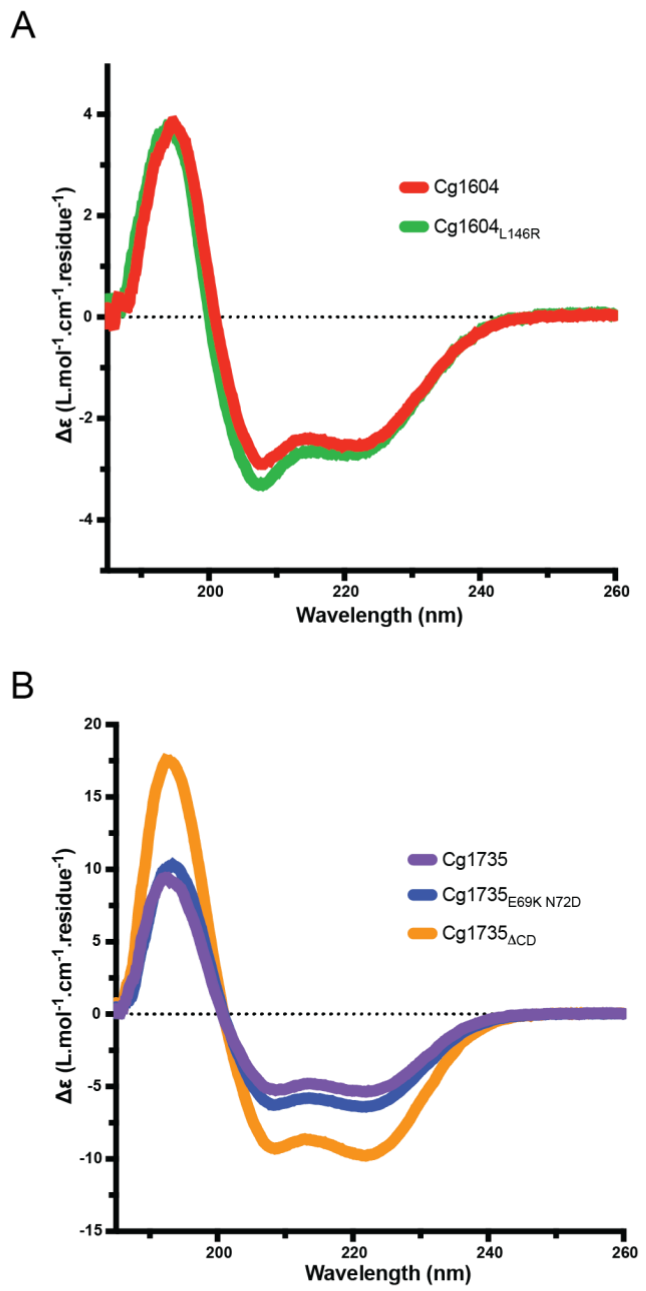

Fig. S11. The circular dichroism profiles of Cg1604 (A) and Cg1735 (B) point mutants are identical to those of the wild-type proteins, indicating that the amino acid substitution introduced no significant conformational change in the protein. As a control, the profile of Cg1735 without the catalytic domain (Cg1735$_{\Delta CD}$) suggests a higher α-helical content, in agreement with the AlphaFold2-predicted structure (Figure S7d).

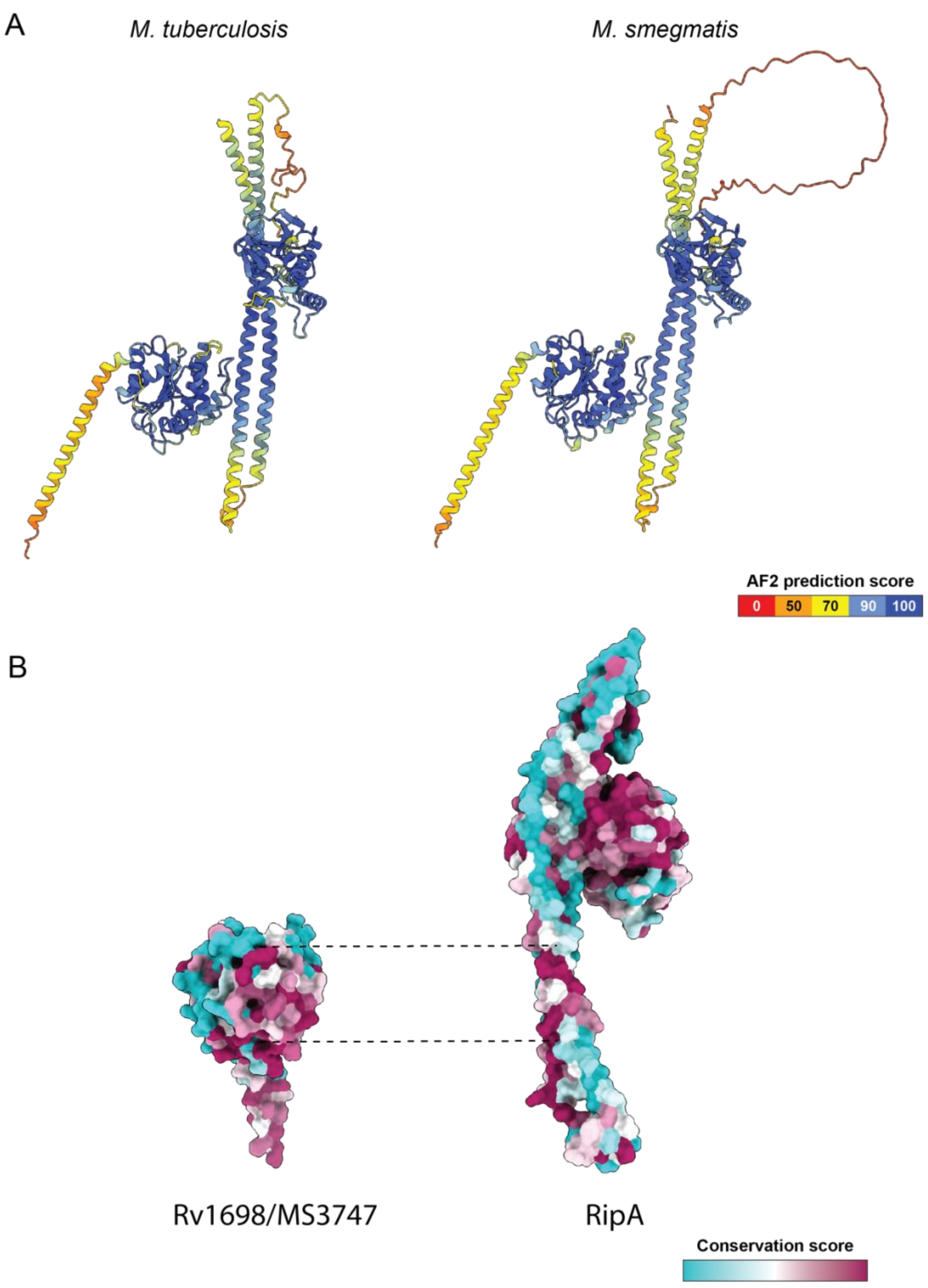

Fig. S12. RipA activation complex in mycobacteria. (A) AlphaFold2-predicted structures of the putative RipA activation complexes in *Mtb* (Rv1698-RipA) and *M. smegmatis* (MS3747-RipA) colored by model confidence. The complex structures are identical to that of the *Cglu* complex Cg1604-Cg1735 (Figure S9). (B) Conservation pattern of the RipA/Rv1698 interfaces in *Mycobacteriaceae* (open book representation).

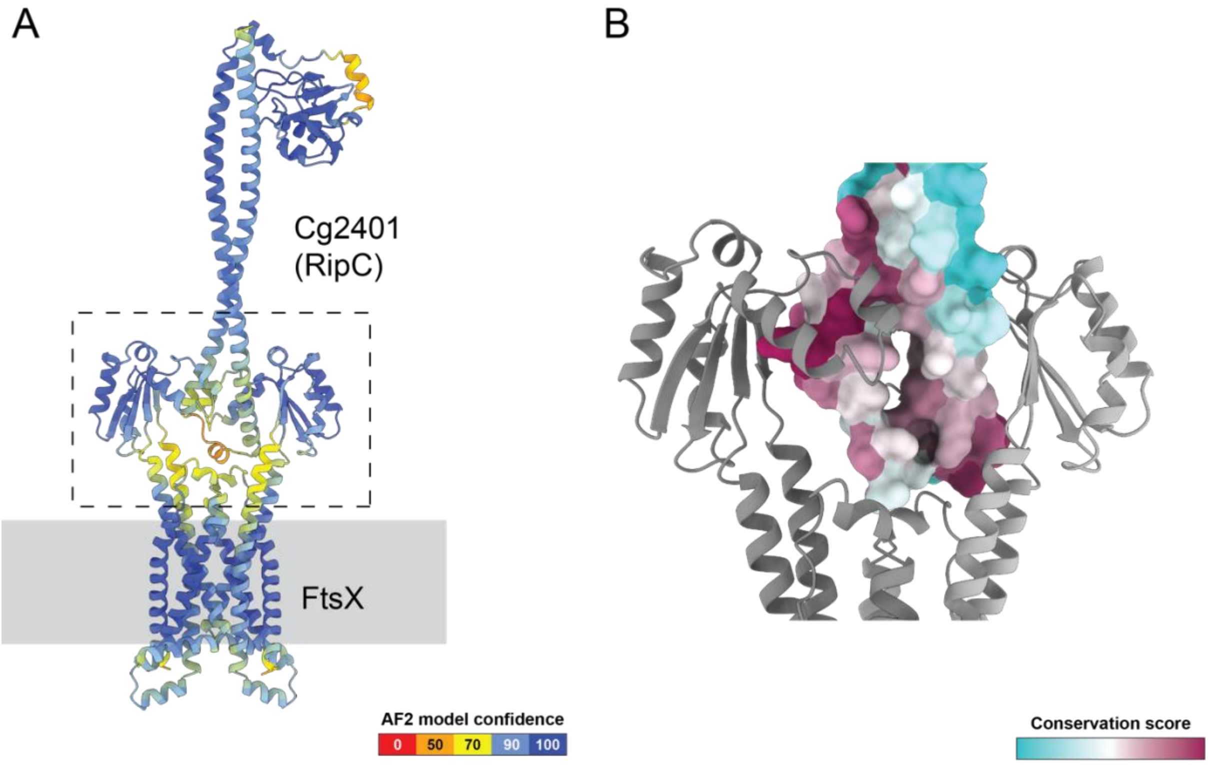

Fig. S13. Model of the Cg2401-FtsX complex. (A) AlphaFold2-predicted model of the putative *Cglu* Cg2401-FtsX complex color-coded according to model confidence. Identical structures are predicted for the homologous RipC-FtsX complexes in *Mtb* and *M. smegmatis* (not shown). (B) Zoom of the molecular interface between Cg2401 (surface representation color-coded according to sequence conservation in the RipC family) and FtsX (cartoon representation).

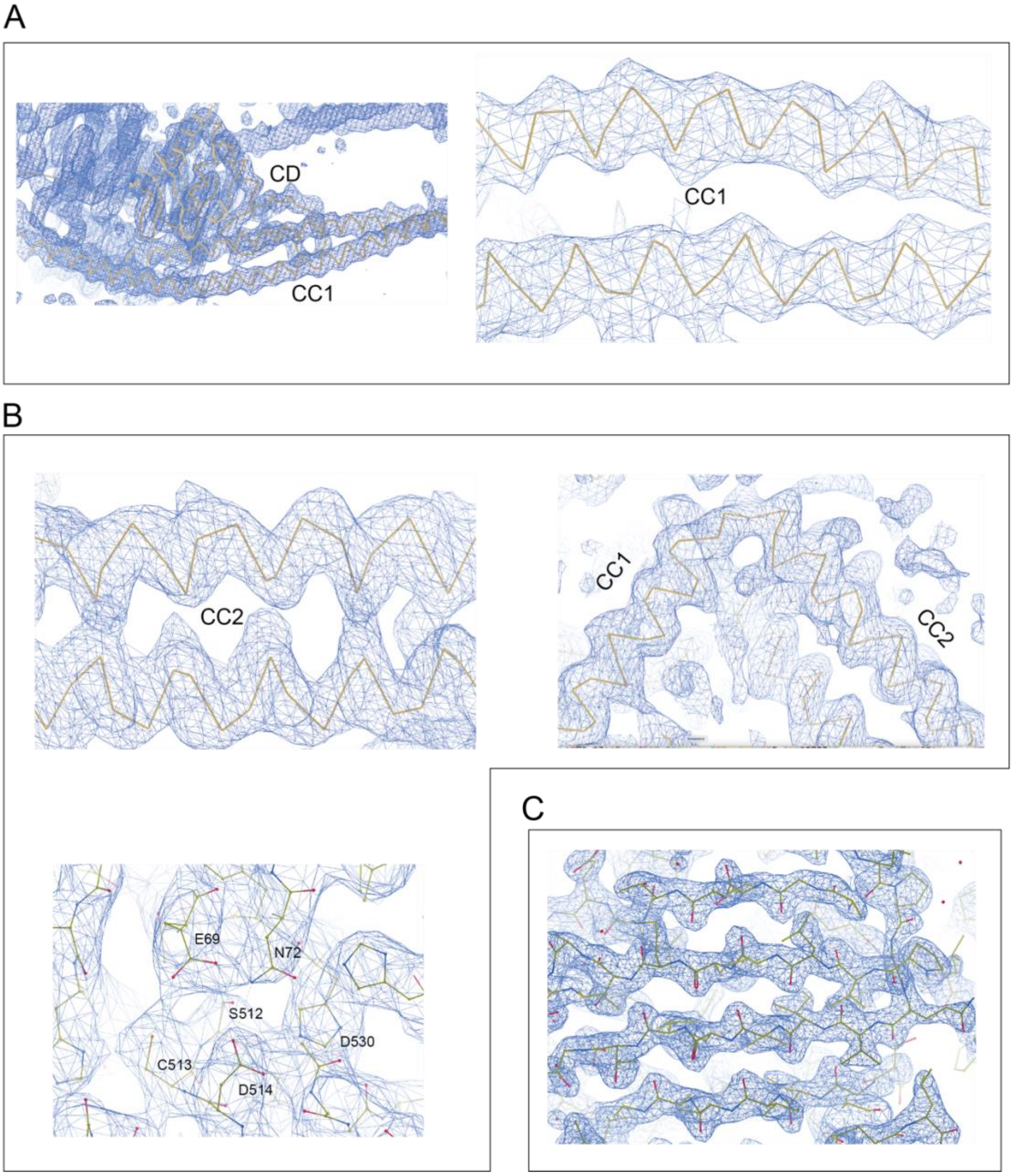

Fig. S14. Representative regions of the final electron density map for the three crystal structures contoured at 1.3 – 1.5 σ, drawn with Coot. (A) Overall view of the orthorhombic structure of Cg1735 at 4.5 Å resolution (left panel) and detail of the CC1 domain (right panel). (B) Detail of the CC2 domain in the trigonal structure of Cg1735 at 3.5 Å resolution (top left), the connecting region between CC1 and CC2 (top right), and close view of the hydrogen bonding interactions between the catalytic residue Cys514 and CC1 residues E69 and N72 (bottom left). (C) Overall view of the central β-sheet of Cg1604 at 2 Å resolution.

Table S1. List of taxa used for the phylogenetic analysis and NlpC/P60 homologs identified in the order *Corynebacteriales* (class *Actinomycetia*, phylum *Actinobacteria*). The NCBI TaxID, taxonomy and NCBI Genbank accession numbers of the homologs are provided.

NCBI Tax_ID	Family	Genus	Species	RipA	RipB	RipC	RipC'	Other_NlpC/P60
196627	Corynebacteriaceae	Corynebacterium	C. glutamicum	BAB98931.1	NA	BAB99580.1	BAB99581.1	BAB98075.1
548476	Corynebacteriaceae	Corynebacterium	C. aurimucosum	ACP32831.1	NA	ACP33287.1	ACP33288.1	ACP32132.1
1408189	Corynebacteriaceae	Corynebacterium	C. lactis	ALA67406.1	NA	ALA67664.1	ALA67665.1	ALA66786.1
1717	Corynebacteriaceae	Corynebacterium	C. diphtheriae	CKG95155.1	NA	CKH05141.1	CKH05162.1	CKG74673.1
1431546	Corynebacteriaceae	Corynebacterium	C. aquilae	APT84782.1	NA	APT85116.1	APT85117.1	NA
499555	Dietziaceae	Dietzia	D. timorensis	NA	NA	NA	ANI92137.1, ANI94021.1, ANI94023.1	ANI91791.1
712270	Dietziaceae	Dietzia	D. sp. oral taxon 368	AVM65987.1	NA	AVM64986.1	AVM64985.1	AVM64740.1
546160	Dietziaceae	Dietzia	D. lutea	AWH93833.1	NA	AWH91838.1	AWH91837.1	AWH91584.1
139021	Dietziaceae	Dietzia	D. psychralcaliphila	AWH97311.1	NA	AWH95147.1	AWH95146.1	AWH94869.1
912801	Dietziaceae	Dietzia	D. sp. DQ12-45-1b	QGW24711.1	NA	QGW24352.1	QGW24350.1	QGW23955.1
526226	Gordoniaceae	Gordonia	G. bronchialis	ACY21645.1	NA	ACY22256.1	ACY20229.1, ACY22257.1	ACY21041.1
1112204	Gordoniaceae	Gordonia	G. polyisoprenivorans	AFA73366.1	NA	AFA73827.1	AFA73828.1	AFA72720.1
1136941	Gordoniaceae	Gordonia	G. phthalatica	ALG84813.1	NA	ALG86863.1	ALG85173.1	ALG84227.1
2420509	Gordoniaceae	Gordonia	G. insulae	AZG44349.1	NA	AZG43671.1	AZG43670.1, AZG45858.1	AZG44885.1
84096	Gordoniaceae	Gordonia	G. alkanivorans	AZZ82179.1	NA	AZZ81759.1	AZZ81758.1	AZZ82893.1
1528099	Lawsonellaceae	Lawsonella	L. clevelandensis	ALE34059.1	NA	NA	NA	NA
443218	Mycobacteriaceae	Hoyosella	H. subflava	AEF40366.1	NA	AEF39741.1	AEF39740.1, AEF40106.1	AEF42303.1
83332	Mycobacteriaceae	Mycobacterium	M. tuberculosis	CCP44237.1	CCP44238.1	CCP44967.1	NA	CCP42746.1, CCP44330.1
1138383	Mycobacteriaceae	Mycobacterium	M. paraintracellulare	NA	AFC54726.1	AFC53674.1	NA	AFC51548.1, AFC54630.1
1131442	Mycobacteriaceae	Mycobacterium	M. marinum	CDM76353.1	CDM76354.1	CDM77279.1	NA	CDM74192.1, CDM76450.1
64667	Mycobacteriaceae	Mycobacterium	M. lepraemurium	ATA28341.1	ATA28342.1	ATA28480.1	NA	ATA28846.1
1769	Mycobacteriaceae	Mycobacterium	M. leprae	AWV48269.1	AWV48268.1	AWV47637.1	NA	AWV49023.1
1069220	Mycobacteriaceae	Mycobacterium	M. koreense	BBY53763.1	BBY53762.1	BBY53285.1	NA	BBY53709.1, BBY55910.1
1578165	Mycobacteriaceae	Mycobacteroides	M. saopaulense	ALR14140.1	ALR14139.1	ALR14009.1	NA	ALR11823.1, ALR12800.1
83262	Mycobacteriaceae	Mycobacteroides	M. immunogenum	AMT71348.1	AMT73835.1	AMT70685.1	NA	AMT71118.1
36809	Mycobacteriaceae	Mycobacteroides	M. abscessus	AMU46167.1	AMU48467.1	AMU45510.1	NA	AMU45950.1, AMU46983.1
1460372	Mycobacteriaceae	Mycobacteroides	M. chelonae	ANA98694.1	ANA98693.1	ANA98064.1	NA	ANA98474.1, ANA99637.1
404941	Mycobacteriaceae	Mycobacteroides	M. salmoniphilum	QCH24166.1	QCH24165.1	QCH23509.1	NA	QCH23935.1, QCH25187.1
875328	Mycobacteriaceae	Mycolicibacter	M. sinensis	AEF36237.1	AEF36236.1	AEF35713.1	NA	AEF34040.1, AEF36164.1
29314	Mycobacteriaceae	Mycolicibacter	M. hiberniae	BBZ23266.1	BBZ23265.1	BBZ22740.1	NA	BBZ23204.1, BBZ25244.1
1118379	Mycobacteriaceae	Mycolicibacter	M. minnesotensis	BBY35776.1	BBY35775.1	BBY35264.1	NA	BBY33731.1, BBY35712.1
1788	Mycobacteriaceae	Mycolicibacter	M. terrae	SNV74975.1	SNV74965.1	SNV69122.1	NA	SNV52932.1, SNV74191.1
350058	Mycobacteriaceae	Mycolicibacterium	M. vanbaalenii	ABM13147.1, ABM13554.1, ABM14444.1	ABM13555.1, ABM14440.1, ABM14474.1	ABM14347.1, ABM14498.1	NA	ABM12428.1, ABM13775.1
1772	Mycobacteriaceae	Mycolicibacterium	M. smegmatis	CKH84184.1	CKH84225.1	CKI12421.1	NA	CKH31193.1, CKH94297.1
1794	Mycobacteriaceae	Mycolicibacterium	M. gadium	BBZ15999.1, BBZ16840.1	BBZ16839.1	BBZ15991.1, BBZ16110.1	NA	BBZ16592.1, BBZ18254.1
28047	Mycobacteriaceae	Mycolicibacterium	M. confluentis	BBZ33906.1	BBZ33905.1	BBZ33171.1	NA	BBZ33582.1, BBZ36608.1
1122247	Mycobacteriaceae	Mycolicibacterium	M. hassiacum	VCT90411.1	VCT90410.1	VCT89819.1	NA	VCT88759.1, VCT90213.1
247156	Nocardiaceae	Nocardia	N. farcinica	BAD58329.1	NA	BAD56566.1	BAD56567.1, BAD56623.1, BAD60447.1	BAD55802.1, BAD58518.1
2213200	Nocardiaceae	Nocardia	N. mangyaensis	APE35657.1	NA	APE38147.1	APE34068.1, APE37799.1, APE38160.1	APE33384.1, APE35770.1
37326	Nocardiaceae	Nocardia	N. brasiliensis	ASF06577.1	NA	ASF08886.1	ASF08885.1, ASF09615.1, ASF11714.1, ASF13030.1, ASF13347.1	ASF06412.1
1823	Nocardiaceae	Nocardia	N. otitidiscaviarum	QDP79872.1	NA	QDP78738.1	QDP78737.1, QDP83121.1, QDP83306.1, QDP83926.1	QDP81622.1, QDP83638.1
1824	Nocardiaceae	Nocardia	N. asteroides	VEG36509.1	NA	VEG34441.1	VEG31843.1, VEG34442.1, VEG34503.1	VEG33511.1, VEG33936.1, VEG36675.1
1136179	Nocardiaceae	Rhodococcus	R. erythropolis	AGT92669.1	NA	AGT93137.1	AGT93138.1, AGT94075.1, AGT94312.1	AGT91840.1, AGT93816.1
1051973	Nocardiaceae	Rhodococcus	R. fascians	AMY55627.1	NA	AMY55228.1	AMY55227.1	AMY51478.1, AMY54298.1, AMY56257.1
334542	Nocardiaceae	Rhodococcus	R. qingshengii	AUS32332.1	NA	AUS32751.1	AUS32752.1, AUS33628.1, AUS33843.1	AUS31493.1, AUS33325.1
1830	Nocardiaceae	Rhodococcus	R. ruber	AWG99201.1	NA	AWG99569.1	AWG99570.1	AWG98213.1, AWH01753.1
300028	Nocardiaceae	Rhodococcus	R. triatomae	QNG21327.1	NA	QNG18503.1	QNG21109.1, QNG21274.1	QNG17974.1, QNG19491.1
640132	Segniliparaceae	Segniliparus	S. rotundus	ADG97938.1, ADG97939.1	NA	ADG97537.1	NA	ADG96668.1
521096	Tsukamurellaceae	Tsukamurella	T. paurometabola	ADG78689.1	NA	ADG79283.1	ADG76677.1, ADG79284.1	ADG77611.1
57704	Tsukamurellaceae	Tsukamurella	T. tyrosinosolvens	AUN40516.1	NA	AUN41111.1	AUN41112.1, AUN42731.1	AUN43155.1
2597660		Tomitella	T. sp. HY188	QDQ99192.1	NA	QDQ97893.1	QDQ97894.1, QDQ99405.1	QDQ98347.1

Table S2. Crystallographic data collection and refinement statistics.

**Data collection**	**Cg1735 Cl_4K_2Pt derivative Trigonal crystal form**	**Cg1735 Orthorhombic crystal form**	**Cg1604 SeMet-labeled**
Synchrotron Beamline	SOLEIL Proxima 1	SOLEIL Proxima 2A	SOLEIL Proxima 1
Wavelength (Å)	1.07188	0.980112	0.979261
Space group	H32	$P2_12_12_1$	$C222_1$
Cell dimensions *a*, *b*, *c* (Å)	230.6, 230.6, 416.7	96.57, 123.08, 209.51	66.78, 105.50, 70.45
Resolution (Å)	49.6 – 3.49 (3.58 – 3.49) *	61.5 – 4.5 (5.03 – 4.5)	44.1 – 2.0 (2.05 – 2.0)
*R*pim	0.049 (7.886)	0.117 (0.342)	0.035 (0.642)
I / s(I)	13.4 (0.4)	5.5 (2.3)	16.1 (1.1)
Completeness (%)	99.6 (98.1)	100 (100)	99.7 (95.9)
CC(1/2)	1.0 (0.52)	0.99 (0.93)	1.0 (0.55)
Multiplicity	115.6 (101.4)	13.5 (13.7)	14.6 (11.5)
Total observations	6263237	207716	250529
Unique observations	54157 (3903)	15441 (4293)	17209 (1207)
**Refinement**			
Resolution (Å)	3.5	4.5	2.
No. reflections	30819 **	11524 **	17156
*R*work/ *R*free (%)	0.226 / 0.260	0.284 / 0.307	0.194 / 0.228
No. atoms			
Protein	7197	5154	1803
Ligands/ions	2 (Pt)	-	1 (Ca)
Solvent	-	-	108
Average B-factors ($Å^2$)			
Protein	89	93	48
Ligand/ions	131	-	76
Solvent	-	-	52
R.m.s deviations			
Bond lengths (Å)	0.012	0.013	0.002
Bond angles (º)	1.502	1.770	0.413
Ramachandran favored (%)	97	94.5	98.8
Ramachandran outliers (%)	0.2	0.6	0
**PDB code**	8AUC	8AUD	8AU6

*Values in parenthesis correspond to the highest resolution shell.
**Number of unique reflections used for structure determination and refinement, after the merged dataset was subjected to anisotropic resolution surface cut-off with StarAniso (see Methods).

Table S3. Plasmids used in this work.

Plasmid	Description
pUMS_215	KanaR; pET derivate containing a N-terminal His-tag followed by a SUMO protease cleavage site
pUMS_216	KanaR; pET derivate for *Cglu* Cg1735 (20-600) recombinant expression with a N-terminal His-tag followed by a SUMO protease cleavage site
pUMS_217	KanaR; pET derivate for *Cglu* $Cg1735_{\Delta CD}$ (20-386) recombinant expression with a N-terminal His-tag followed by a SUMO protease cleavage site
pUMS_218	KanaR; pET derivate for *Cglu* $Cgl1735_{CC1}$ (20-238) recombinant expression with a N-terminal His-tag followed by a SUMO protease cleavage site
pUMS_219	KanaR; pET derivate for *Cglu* $Cg1735_{CD}$ (460-600) recombinant expression with a N-terminal His-tag followed by a SUMO protease cleavage site
pUMS_220	KanaR; pET derivate for *Cglu* Cg1604 (36-295) recombinant expression with a N-terminal His-tag followed by a SUMO protease cleavage site
pUMS_221	KanaR; pET derivate for *Cglu* $Cg1604_{L146R}$ (36-295, carrying amino acid substitution L146R) recombinant expression with a N-terminal His-tag followed by a SUMO protease cleavage site
pUMS_222	KanaR; pET derivate for *Cglu* $Cg1735_{E69K,N72D}$ (20:600, carring amino acid substitutions E69K and N72D) recombinant expression containing a N-terminal His-tag followed by a SUMO protease cleavage site

Table S4. Oligonucleotide primers used in this work.

Name	Sequence (5' to 3') and properties[a]
Construction of pUMS_216	
QG_90	TAAAAGGGCGAGCTCAGATCCGG
QG_91	ACCACCAATCTGCTCACGATGTGC
QG_162	**ATCGTGAGCAGATTGGTGGT**CAGCCACAGAATCCGGATGACG
QG_163	**GATCTGAGCTCGCCCTTTTA**CTAAATGAG GCGTACCACGCTCTC
Construction of pUMS_217	
QG_90	TAAAAGGGCGAGCTCAGATCCGG
QG_91	ACCACCAATCTGCTCACGATGTGC
QG_162	**ATCGTGAGCAGATTGGTGGT**CAGCCACA GAATCCGGATGACG
QG_164	**GATCTGAGCTCGCCCTTTTA**CTGGCTCGC CGCAATTAGAGC
Construction of pUMS_218	
QG_178	CAGATTGGTGGTGATCGTTCCGCTCAGATTGAGACTG
QG_184	CTCGCCCTTTTAATCCGCCTGCGCACGAGCG
Construction of pUMS_219	
QG_178	CAGATTGGTGGTGATCGTTCCGCTCAGATTGAGACTG
QG_176	ACCACCAATCTGCTCACGATGTG
Construction of pUMS_220	
QG_90	TAAAAGGGCGAGCTCAGATCCGG
QG_91	ACCACCAATCTGCTCACGATGTGC
QG_94	**ATCGTGAGCAGATTGGTGGT**GAAAACATTGACCCAAATGCACCAACATCAGC
QG_95	**GATCTGAGCTCGCCCTTTTA**CCTACCGGC TAGTTCCTCACGAACTG
Construction of pUMS_221	
QG_185	CAGC**G**TTCTGAAACCCAACTGGATCCAGG
QG_186	TTCAGAA**C**GCTGAGCGCCTGCAGG
Construction of pUMS_222	
QG_187	**A**AAGAAGTG**G**ACAAGTCCCTCGTGGATTTGCAT
QG_188	GT**C**CACTTCTT**T**ACGCAGAGCACCCATTTC

[a] Overlaps for Gibson assembly are in bold letters, mutated positions are in red.
